# Agronomic, physiological and transcriptional characteristics provide insights into fatty acid biosynthesis in yellowhorn (*Xanthoceras sorbifolium* Bunge) during fruit ripening

**DOI:** 10.3389/fgene.2024.1325484

**Published:** 2024-01-31

**Authors:** Guan Liu, Fengjiao Liu, Lin Pan, Hanhui Wang, Yanan Lu, Changhua Liu, Song Yu, Xiaohang Hu

**Affiliations:** ^1^ State Key Laboratory of Tree Genetics and Breeding, College of Forestry, Northeast Forestry University, Harbin, China; ^2^ College of Advanced Agriculture and Ecological Environment, Heilongjiang University, Harbin, China; ^3^ Key Laboratory of Saline-alkali Vegetation Ecology Restoration, Ministry of Education, College of Life Science, Northeast Forestry University, Harbin, China

**Keywords:** *Xanthoceras sorbifolia* Bunge, growth and development, physiological indicators, transcriptome sequencing, fatty acid biosynthesis

## Abstract

Yellowhorn (*Xanthoceras sorbifolium* Bunge) is an oil-bearing tree species in northern China. In this study, we used yellowhorn from Heilongjiang to analyze the morphological and physiological changes of fruit development and conducted transcriptome sequencing. The results showed that the fruit experienced relatively slow growth from fertilization to DAF20 (20 days after flowering). From DAF40 to DAF60, the fruit entered an accelerated development stage, with a rapid increase in both transverse and longitudinal diameters, and the kernel contour developed completely at DAF40. From DAF60 to DAF80, the transverse and vertical diameters of the fruit developed slowly, and the overall measures remained stable until maturity. The soluble sugar, starch, and anthocyanin content gradually accumulated until reaching a peak at DAF80 and then rapidly decreased. RNA-seq analysis revealed differentially expressed genes (DEGs) in the seed coat and kernel, implying that seed components have different metabolite accumulation mechanisms. During the stages of seed kernel development, *k*-means clustering separated the DEGs into eight sub-classes, indicating gene expression shifts during the fruit ripening process. In subclass 8, the fatty acid biosynthesis pathway was enriched, suggesting that this class was responsible for lipid accumulation in the kernel. WGCNA revealed ten tissue-specific modules for the 12 samples among 20 modules. We identified 54 fatty acid biosynthesis pathway genes across the genome, of which 14 was quantified and confirmed by RT-qPCR. Most genes in the plastid synthesis stage showed high expression during the DAF40–DAF60 period, while genes in the endoplasmic reticulum synthesis stage showed diverse expression patterns. EVM0012847 (KCS) and EVM0002968 (HCD) showed similar high expression in the early stages and low expression in the late stages. EVM0022385 (HCD) exhibited decreased expression from DAF40 to DAF60 and then increased from DAF60 to DAF100. EVM0000575 (KCS) was increasingly expressed from DAF40 to DAF60 and then decreased from DAF60 to DAF100. Finally, we identified transcription factors (TFs) (HB-other, bHLH and ARF) that were predicted to bind to fatty acid biosynthesis pathway genes with significant correlations. These results are conducive to promoting the transcriptional regulation of lipid metabolism and the genetic improvement in terms of high lipid content of yellowhorn.

## 1 Introduction

As a relic oil-bearing woody plant, *Xanthoceras sorbifolium* Bunge (yellowhorn) (2n = 30) is the only species in its genus (Yang et al., 2005). Yellowhorn is a deciduous undershrub or small tree (Supplementary Figure S1) and is a native woody species that is widely cultivated in the arid and semiarid areas of northwestern China (Zhang and Zhou, 2013). Yellowhorn can withstand extreme cold (−40°C), drought (Ruan et al., 2017; Jin et al., 2020; Liu et al., 2020), salt and barren terrain and has strong resistance to pests and diseases (Venegas-Calerón et al., 2017; Wang et al., 2018a). It has a long cultivation history and has multiple functions, such as in landscaping, with high economic and ecological value (Wang et al., 2017; Lang et al., 2020). The fruit of yellowhorn is rich in unsaturated fatty acids (Ruan et al., 2017), which can be used to make high-grade vegetable oils with neuroprotective, antitumor, strong antioxidant and free radical scavenging effects (Zang et al., 2021; Chen et al., 2022) and can also serve as an important source of bioenergy as a substitute for petroleum diesel fuel (Zhang et al., 2016). The fruit is also rich in protein, containing 18 amino acids, vitamin A, and vitamin C (Liang et al., 2021). In addition, yellowhorn has medicinal value. The flavonoids in the fruit have anti-inflammatory and anti-rheumatic effects; the myricetin in the leaves has antibacterial, vascular stabilizing, and hemostatic effects; the coptisine in the calyx has antipyretic and hypnotic effects; and the saponin E in the fruit shell has a marked effect on improving memory function (Ruan et al., 2017; Chen et al., 2022).

The seeds of yellowhorn contain 19 types of fatty acids, and the content of the major oil-producing tissue, the seed kernel, is 55%–70% oil, of which the dominant component is unsaturated fatty acids (94%) (Yu et al., 2017), such as linoleic acid and oleic acid (Venegas-Calerón et al., 2017). In particular, the content of nervonic acid, a relatively rare fatty acid that is indispensable for nervous system development, was found to be 3.80% (Liang et al., 2021). The ratio of saturated fatty acids: monounsaturated fatty acids: polyunsaturated fatty acids in yellowhorn oil was closest to the ideal ratio (1:1:1), indicating a balanced nutrient distribution.

Fatty acids in oil bodies are primarily stored in the form of triglycerides, which includes glycerol and fatty acids. Seed fatty acids are synthesized in the plastid with a diverse range of forms, and their degree of unsaturation is influenced by chain length. The polymerization reaction occurs in two stages. The first step in the formation of plant oil is the synthesis of fatty acids, which is catalyzed by acetyl-CoA carboxylase (ACC) to produce malonyl-CoA from acetyl-CoA. Then, enzymes such as malonyl-CoA ACP transacylase (MCMT), β-ketoacyl-ACP synthase (KAS) series enzymes, 3-ketoacyl-CoA reductase (KAR), fatty acid synthase (FASN), and fatty acyl-ACP thioesterase (FAT) catalyze the formation of free fatty acids (FFAs) of different lengths, resulting in the production of C16- and C18-saturated fatty acids. FFAs are catalyzed by acyl-CoA synthetase (LACS) to form acyl-CoA. After FFAs of different lengths are converted into acyl-CoA, they are transported to the cytoplasm and endoplasmic reticulum. Acyl CoA and 3-phosphatidylglycerol can then be converted into triacylglycerol (TAG) by enzymes including 3-ketoacylCoA synthase (KCS), 3-ketoacyl-CoA reductase (KCR), 3-hydroxacyl-CoA dehydratase (HCD), and trans-2,3-enoyl-CoA reductase (ECR), resulting in the production of the C18, C20, C22, and C24 fatty acid series (Liang et al., 2022).

Plant oil synthesis is a complex process involving multiple enzymatic reactions. Exploring and analyzing the genes and regulatory mechanisms related to oil synthesis has reference significance for the comprehensive elucidation of the oil synthesis metabolic regulatory network. Currently, there are some reports on fatty acid synthesis genes related to *Xanthoceras sorbifolia*. Some functionally characterized genes associated with fatty acid biosynthesis in yellowhorn, such as XSDGAT, XsFAD2, XsFAE1, XsHCD, XsGPD1, XsDGAT1, XSDGAT2, XsLEC1 and XsSAD, have been well summarized (Lang et al., 2020). Recently, Liang et al. (2019) identified a 3-ketoacyl-CoA synthase gene (XS04G00959) by integrating yellowhorn genomic and transcriptomic data whose expression was in line with the accumulation of nervonic and erucic acid, indicating its potential roles in the biosynthesis process. The miRNA-target regulatory modules miR319p_1-FAD2-2 (omega-6 fatty acid desaturase 2-2) and miR5647-p3_1-DGAT1 (diacylglycerol acyl-transferase 1), which may participate in fatty acid biosynthesis, have been identified in yellowhorn (Wang et al., 2021). Subsequently, in extended regulatory modules that included lncRNA, KCS11-1-miR156g-2LNC_000849 and DGAT-2-miR172j-LNC_005874 were found to participate in nervonic acid synthesis and TAG accumulation (Hong et al., 2023).

Research progress on the genomes of woody oilseed plants has lagged behind that on herbaceous oil crops. In recent years, due to technological development in sequencing and the reduction of sequencing costs, there has been some progress in sequencing the genomes of woody oilseed plants, such as olive (*Olea europaea*) and tung tree (*Vernicia fordii*). Important progress in yellowhorn genomics has also been made. Bi et al. (2019) and Liang et al. (2019) simultaneously completed the *X*. *sorbifolium* genome sequencing and assembly, identifying 15 yellowhorn pseudochromosomes, with the genome sizes of the WF18 and hongshi4 cultivars being 439.97 Mb and 504.2 Mb, respectively. Subsequently, Liu et al. (2021) assembled the Jinguanxiapei cultivar genome, which had a size of 470 Mb, and analyzed the transcriptomes from different tissues, identifying candidate genes involved in very-long-chain fatty acid biosynthesis and their expression profiles. Liang et al. (2022) also upgraded the genome assembly for WF18, producing an updated version with a size of 490.44 Mb. These advancements lay a foundation for functional genomics research on *X*. *sorbifolium*, particularly by providing new insights for the mining of functional genes and the development of new yellowhorn resources.

Aside from genome assembly, most related omics research on yellowhorn focuses on gene expression and metabolomics in relation to tissue development and stress response. Wang et al. (2018b) sampled high- and low-oil yellowhorn embryo tissues at four developmental stages for RNA-seq and identified significantly differentially expressed genes (DEGs) involved in fatty acid (FA) biosynthesis, glycolysis/gluconeogenesis, and pyruvate metabolism between the high- and low-oil yellowhorn lines. In addition, several transcription factors, such as AP2-EREBP family members, B3 domain proteins and C2C2-Dof proteins, were identified. Metabolomic and transcriptomic profiling of flowering phases revealed flavonoid accumulation in yellowhorn flowers during their development. The researchers found that samples from the full flowering phase had the highest flavonoid content, among which the content of peonidin-3-O-glucoside was the highest. In addition, the transcriptomic results showed that MYB and WD40 transcription factors might function in anthocyanin accumulation (Wang et al., 2022). Transcriptome sequencing of the four yellowhorn cultivars under long-term drought conditions yielded DEGs whose enrichment occurred in key biological processes and metabolic pathways involved in drought resistance. Coexpression analysis has revealed protein kinase hub genes and coexpressed gene modules associated with water use efficiency (WUE) in yellowhorn (Liu et al., 2020). In another study, on the basis of transcriptomic and metabolomic analyses of yellowhorn seedlings under low-temperature stress, DEGs and differentially expressed metabolites (DEMs) were screened, and conjoint analysis revealed that amino acid metabolism and sugar metabolism were enhanced (Wang et al., 2020a). Similarly, the transcriptional response of yellowhorn to salt stress was also explored. The DEGs expressed in response to salt and saline-alkaline treatments were enriched in carbon metabolism, biosynthesis of amino acids, starch and sucrose metabolism and reactive oxygen species signaling networks. The regulatory role of intracellular pH in coping with saline-alkaline stress has been highlighted (Wang et al., 2020b).

Compared to research in other plant species, the investigation of yellowhorn has been relatively limited, especially regarding the systematic study of fruit development and fatty acid biosynthesis. In this study, we used the fruits of yellowhorn grown in Heilongjiang Province, a high-latitude region in China, at five different developmental stages for morphological, cytological, physiological, and transcriptomic analysis. Our aim was to explore the agronomic characteristics and dynamic developmental patterns of the seed coat and kernel at different developmental stages, as well as the key genes involved in fatty acid synthesis pathways. Our findings will provide a reference for oil-target molecular breeding and gene function mining in yellowhorn.

## 2 Materials and methods

### 2.1 Plant material source and sampling

The experimental materials used in this study were collected from 10 yellowhorn trees with a tree age of approximately 10 years on the campus of China Northeast Forestry University (located at 126°E and 45°N). Based on the days after flowering (DAF), samples were collected for the first time at 20 DAF and then every 20 days after (Supplementary Table S1) for a total of five different developmental stages (20 DAF, 40 DAF, 60 DAF, 80 DAF, and 100 DAF). At each sampling time, fruits were collected from the same directions of the tree, and the seeds were immediately dissected. The seed coat and kernel were separated, quickly frozen in liquid nitrogen, and stored at −80°C for future experiments.

### 2.2 Agronomic trait and physiological material characterization of fruit during development

The fruits, seeds, and kernels were weighed using an electronic balance to calculate the fresh weight at each sample time point. A Vernier caliper was used to measure the transverse and longitudinal diameters of the fruits, seeds, and kernels at different stages. We used Schiff periodic acid-Schiff (PAS) staining (Lyon et al., 2002), naphthol yellow S staining (Ma et al., 2022), and Nile red staining (Greenspan et al., 1985) to stain the starch grains, protein, and fatty acid substances in the seeds, respectively. The soluble protein content was determined using the Coomassie Brilliant Blue G-250 method (Neuhoff et al., 1988). The soluble sugar and starch content were determined using the anthrone colorimetric method (Clegg, 1956). The total anthocyanin content was determined by a conventional spectrophotometric method at 520 nm (Moreno et al., 2005). All the above tests were conducted with six biological replicates.

### 2.3 Trait data processing and analysis

Excel software 2021 (Microsoft, Redmond, WA, USA) was used to input and organize the trait-related data, and SPSS version 22 (SPSS, Chicago, IL, USA) was used for data statistical analysis and multiple comparisons (Duncan) (*p* < 0.05).

### 2.4 Transcriptome sequencing and gene expression analysis

The tissue samples were subjected to total RNA extraction, and agarose gel electrophoresis was used to analyze their integrity. The total RNA quantity required for library construction was 1 μg, with a concentration of ≥50 ng/μL and an OD260/280 range of 1.8–2.2. After quality control, magnetic beads containing oligo(dT) were used to capture the polyA tail mRNA. Double-stranded cDNA was synthesized using reverse transcriptase, and sequencing adapters were added. The resulting product was separated, purified, and screened using PCR amplification and purification to obtain the final library, followed by sequencing on an Illumina NovaSeq 6000 platform (Majorbio Company, Shanghai, China). The output low-quality sequence data were filtered out using the software fastp (Chen et al., 2018) according to the following steps: I) sequences containing adapter sequences or reads that did not insert fragments due to adapter self-ligation were removed; II) the low-quality bases at the end (3′) of the sequence were trimmed, and if bases with a quality score of less than 10 remained in the remaining sequence, the entire sequence was deleted or retained; and III) sequences containing N (module base) were removed. IV) Sequences shorter than 30 bp were deleted. The clean reads were uploaded to SRA database (Sequence Read Archive) in NCBI (PRJNA923394). First, the clean reads were then aligned to the reference genome (Bi et al., 2019) using HISAT2 (Kim et al., 2019), and the corresponding mapped reads were assembled and quantified using StringTie (Pertea et al., 2015). Genes with a TPM value greater than 1 were considered expressed. After obtaining the quantitative data for the genes, principal component analysis (PCA) (Wickham, 2011) and correlation clustering were performed to test the sequencing quality of all the samples. DESeq2 (Anders and Huber, 2010) was used to calculate the differentially expressed genes (DEGs). Functional annotations and enrichment analyses were performed using GO (Dimmer et al., 2012) and KEGG (Kanehisa and Goto, 2000). The transcription factors (TFs) of all DEGs were annotated according to PlantTFDB 4.0 (Jin et al., 2016). The correlation between transcription factors was calculated using the rcorr function of the R language Hmisc software package (Harrell and Harrell, 2019), and a correlation clustering heatmap was plotted using the heatmap software package (Kolde, 2015). The *k*-means clustering algorithm was used to classify the above DEGs. The gap statistic computation with R was adopted to assess the numbers of subclasses and the goodness of clustering (Tibshirani et al., 2001). For WGCNA, all the expressed genes (TPM>1) of seed kernels were isolated as a base gene set. A gene coexpression network was built using the WGCNA package (Version 4.0.2) (Langfelder and Horvath, 2008). The parameters were set as power = 12, minModuleSize = 30, and MEDissThres = 0.25.

### 2.5 Identification of fatty acid-associated genes in *X*. *sorbifolium*

To identify fatty acid-associated genes, we downloaded the protein sequences of reported fatty acid-associated genes from the NCBI databases. These protein sequences were used as query sequences to search against protein sequences of *X*. *sorbifolium*, and the putative proteins were obtained by BLASTP search with parameters “E-value ≤1 × 10−20, bit score ≥200 and alignment identity ≥30%”. In addition, putative proteins without the same Pfam domains as the query genes were excluded from the analysis.

### 2.6 Reverse-transcription quantitative PCR (RT-qPCR) assay to confirm the expression of selected genes

A total of 14 genes were selected for RT-qPCR assay. The primers were designed by primer3 (https://bioinfo.ut.ee/primer3-0.4.0/) (Supplementary Table S2). The young leaves of three trees were sampled for total RNA extraction using the same method as that described above for RNA-seq. RT-qPCR was performed on a Roche Light Cycler 96 (Roche, Switzerland) using Actin as an internal reference. The PCR system was as follows: Power SYBR^®^ Green PCR Master Mix (Applied Biosystems, Foster City, CA, USA) 10 μL, forward primer (10 μM) 1 μL, reverse primer (10 μM) 1 μL, cDNA template 1 μL, nuclease-free H2O 2 μL. The reaction conditions referred to the method of Liu et al. (2018). The relative expression level of the target gene was determined by the 2^−⊿⊿CT^ method (Livak and Schmittgen, 2001).

## 3 Results

### 3.1 Morphological development dynamics of yellowhorn fruit

**FIGURE 1:**
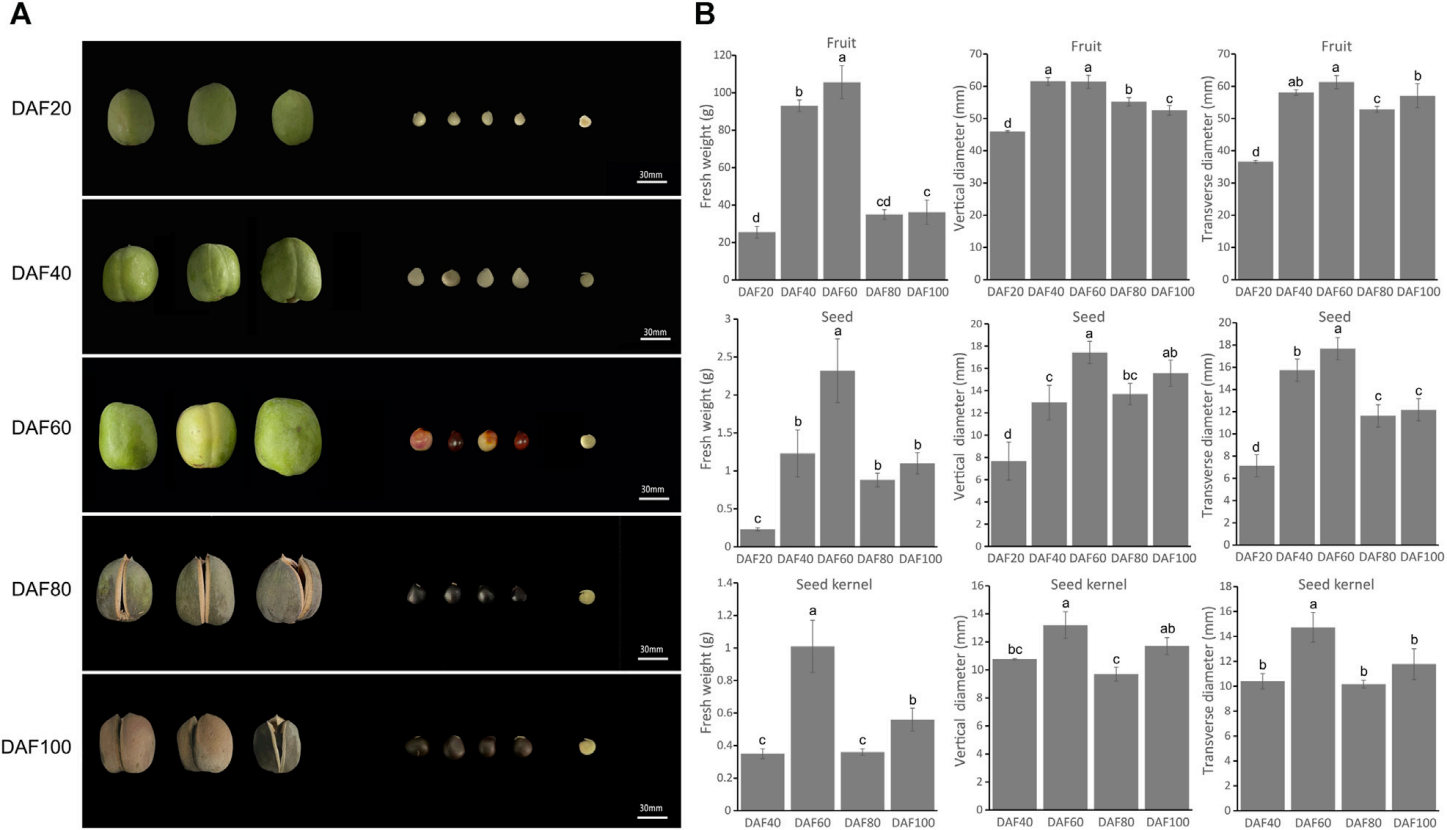
Morphological characteristics of yellowhorn fruits, seeds and kernels. **(A)** The perfor-mance of fruit, seed and kernel at five key different development stages. **(B)** The histograms indicate the fresh weight of fruit, seed and seed kernel at different stages. The weight measurements included seven biological replicates, and multiple comparisons were performed using Duncan’s multiple range test (*p* < 0.05).

The size and color of the fruit, seeds, and kernels of yellowhorn undergo significant changes during fruit development. Samples of five key fruit development stages were therefore collected: DAF 20, DAF 40, DAF 60, DAF 80, and DAF 100 (Figure 1A). The fruit skin gradually changed from the initial tender green at DAF20 to yellow, then dark green, and finally at the maturation stage changed to dark brown (DAF100) with a harder texture. The seed coat also changed from the initial white to red with black patches until it turned completely dark brown. From DAF 20 onwards, the size of the fruit and seeds gradually increased. Notably, the seed coat had not fully developed at DAF 20, so the seed kernel could not be observed in the longitudinal section. At DAF40, the seed kernel was clearly observed, and it gradually increased in size and turned from a delicate green to a creamy white. The seed coat gradually became thinner, and at DAF100, the seed kernel color changed to light yellow.

The trait statistical data indicated that the fresh weight, transverse diameter, and longitudinal diameter of the fruit, seeds, and kernels reached their maximum values at DAF 60. At this stage, the fruit, seeds, and kernels of yellowhorn grew rapidly in both the transverse and longitudinal dimensions. At DAF60, the transverse and longitudinal diameters of the fruit reached their maximum values of 61.40 mm and 61.27 mm, respectively (Figure 1B), which were significantly higher than those at other stages. Subsequently, there were varying degrees of decline from DAF60 to DAF80, and the fresh weight and vertical diameter significantly increased again from DAF80 to DAF100, while the transverse diameter did not significantly increase.

### 3.2 Determination of inclusion content

**FIGURE 2:**
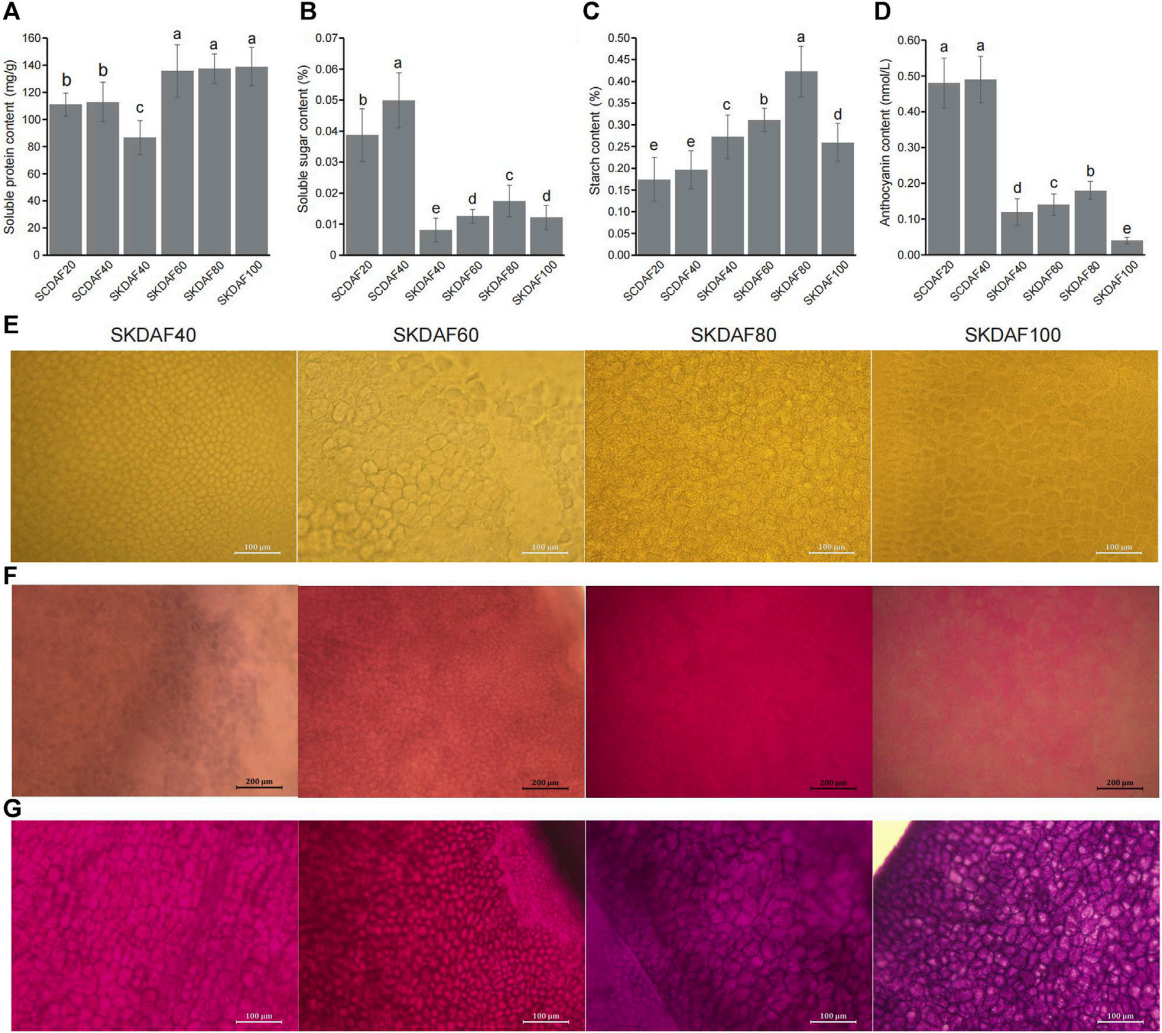
Determination of soluble protein, soluble sugar, starch and anthocyanin contents in the seed coat and kernel of yellowhorn. **(A–D)** Indicate the content of soluble protein, soluble sugar, starch and anthocyanin during the development of the seed coat (SCDAF20 and SCDAF40) and seed kernel (SKDAF40, SKDAF60, SKDAF80 and SKDAF100), respectively. **(E–G)** Indicate naphthol yellow S staining, Nile red staining and PAS staining in seed kernels at stages SKDAF40, SKDAF60, SKDAF80 and SKDAF100, respectively. Pro indicates protein; Lip indicates lipid; Star indicates starch.

As shown in Figure 2A, the protein content in the seed coat increased slightly from DAF20 to DAF40, and the protein content in the four stages of the seed kernel showed a significant increase (from SKDAF40 to SKDAF60) followed by a flattening trend. From SKDAF40 to SKDAF60, the soluble protein content in the seed kernel rapidly accumulated and reached its maximum value at SKDAF60. After SKDAF60, the soluble protein content decreased but not significantly. Naphthofuchsine yellow S staining showed that the protein distribution density in the seed endosperm cells increased overall with development, and obvious protein particles were observed at SKDAF40 (Figure 2E). In the soluble sugar content measurement of the five stages of yellowhorn seeds (Figure 2B), the soluble sugar content in the seed coat was much higher than that in the seed kernel overall, and the soluble sugar content at SCDAF40 was significantly higher than that at SCDAF20. The soluble sugar content in the seed kernel displayed an overall increasing trend, and the rate of increase slowed down from SKDAF60 to SKDAF80 compared to the previous stage. At SKDAF100, the soluble sugar content started to decrease and was at the same level as that at SKDFA60. Unlike the soluble sugar content, the starch content in the seed coat did not change significantly during the SCDAF20 and SCDAF40 periods, and the starch content was significantly higher than that in the seed kernel (Figure 2C). The trend of starch content accumulation in the seed kernel was similar to that of soluble sugar, but the starch content was much higher than that of soluble sugar. At SKDAF40, the starch content in the seed kernel was approximately 0.27%, indicating that starch had been synthesized during early development. The starch content in the seed kernel continued to increase and reached its maximum at SKDAF80, after which the starch content rapidly decreased, suggesting that starch might be converted to other substances. PAS staining showed that purple-red starch grains existed in the seed coat cells at the early development stage, and there were dyed purple-red starch grains in the endosperm cells throughout the entire seed development stage (Figure 2G). The anthocyanins in the yellowhorn seed coat were mainly present in the seed coat, and the anthocyanin content of the seed coat was significantly higher than that in the seed kernel. The anthocyanin content in the seed kernel also increased slowly with growth and development and significantly decreased to the lowest value at SKDAF100 (Figure 2D). The accumulation of fatty acids in seeds was observed using Nile red staining. The results showed that the staining color became lighter during the early stage of seed development, indicating that the fatty acid content was relatively low; then, the color gradually deepened to SKDAF80 (Figure 2F).

### 3.3 Transcriptome sequencing and expression of seed coat and kernel development stages in yellowhorn

**FIGURE 3:**
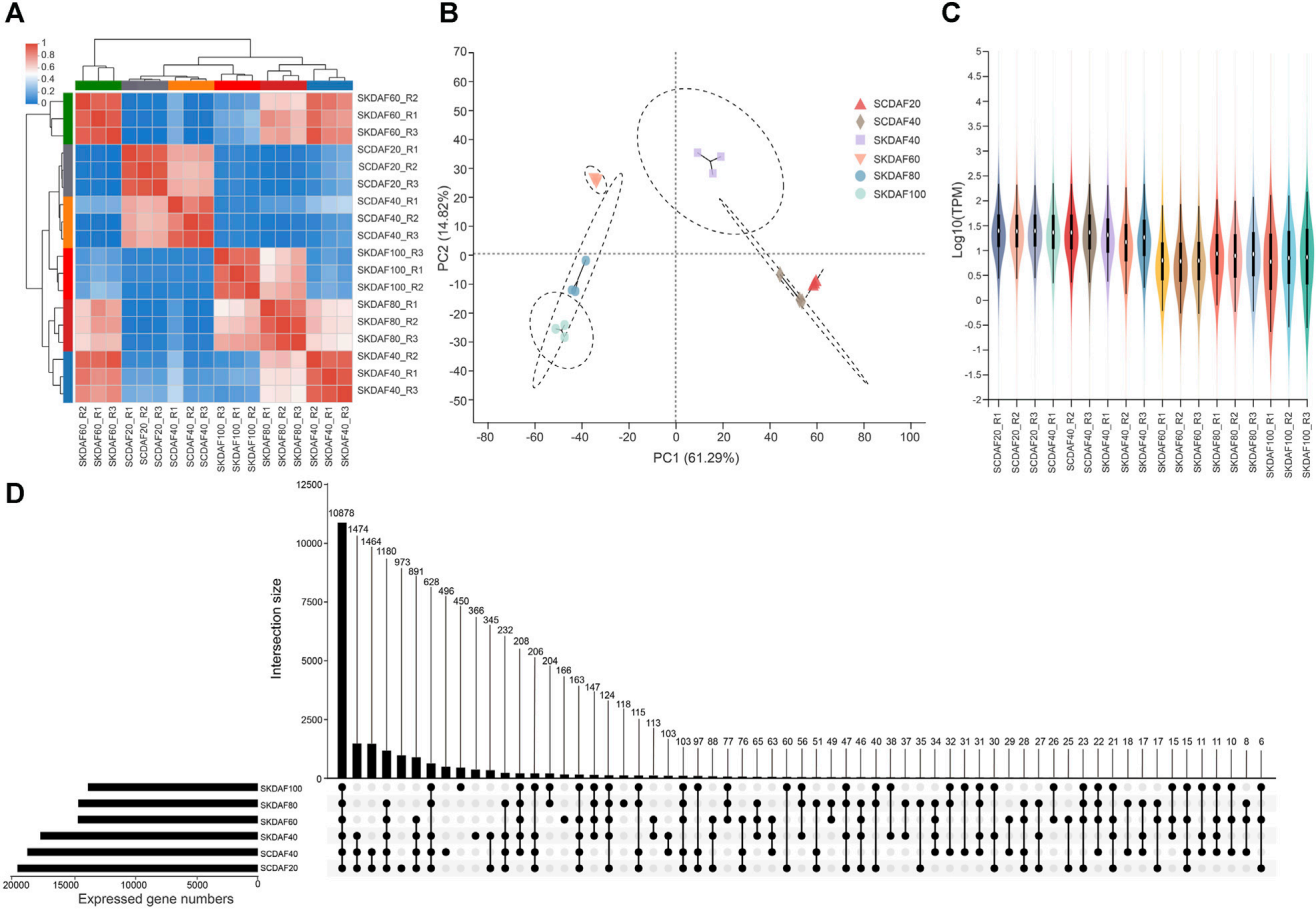
RNA-seq and gene expression statistics in seed coat (SC) and seed kernel (SK). **(A)** Ex-pression correlations among samples. R1, R2, and R3 indicate three biological replicates. **(B)** Principal component analysis (PCA) of 18 different samples. **(C)** Violin map of gene expression. Different colors represent different samples. The width of each violin reflects the number of genes at that level of expression. **(D)** UpSet plot of the expressed genes. The straight line and number indicate gene numbers of the corresponding interaction among samples SKDAF100, SKDAF80, SKDAF60, SKDAF40, SCDAF40 and SCDAF20.

This study included transcriptome sequencing that was conducted at two developmental stages of the seed coat (SCDAF20 and SCDAF40) and four developmental stages of the seed kernel (SKDAF40, SKDAF60, SKDAF80, and SKDAF100), with three biological replicates for each stage. A total of 135.19 Gb clean data were obtained from the transcriptome sequencing, with each sample containing at least 6.56 Gb and a Q30 base percentage of over 93%. After alignment to the reference genome of yellowhorn, the total mapped reads were over 93% for all samples, and the properly mapped reads accounted for a proportion ranging from 78.29% to 85.51% (Supplementary Table S3). Transcripts per million (TPM) were used to quantify gene expression levels for all samples. PCA and correlation clustering were performed on the expression levels, demonstrating that the three biological replicate samples could be clustered into one group (Figures 3A, B). The correlation between the RT-qPCR expression levels of 19 randomly selected genes and their TPM values was 0.8521 (Supplementary Figure S2), further indicating the high quality of the RNA-seq data. The violin plots showed the distribution and probability density of gene expression of different samples, demonstrating good repeatability within each sample group (Figure 3C). An UpSet plot was created to identify the overlapping genes in all SC and SK samples. As shown in Figure 3D, SCDAF20 had the highest number of detected genes, with 19508, while SKDAF100 had the lowest number of detected genes (13938). A total of 10268 genes were shared in all samples. Additionally, 659 genes were exclusively expressed in SCDAF20, while 333 genes were exclusively expressed in SCDAF40. Furthermore, 241, 157, 70, and 58 genes were found to be specifically expressed in SKDAF40, SKDAF100, SKDAF60, and SKDAF80, respectively.

### 3.4 Analysis of differentially expressed genes (DEGs) in comparisons of SCDAF20 vs. SCDAF40 and SCDAF40 vs. SKDAF40

**FIGURE 4:**
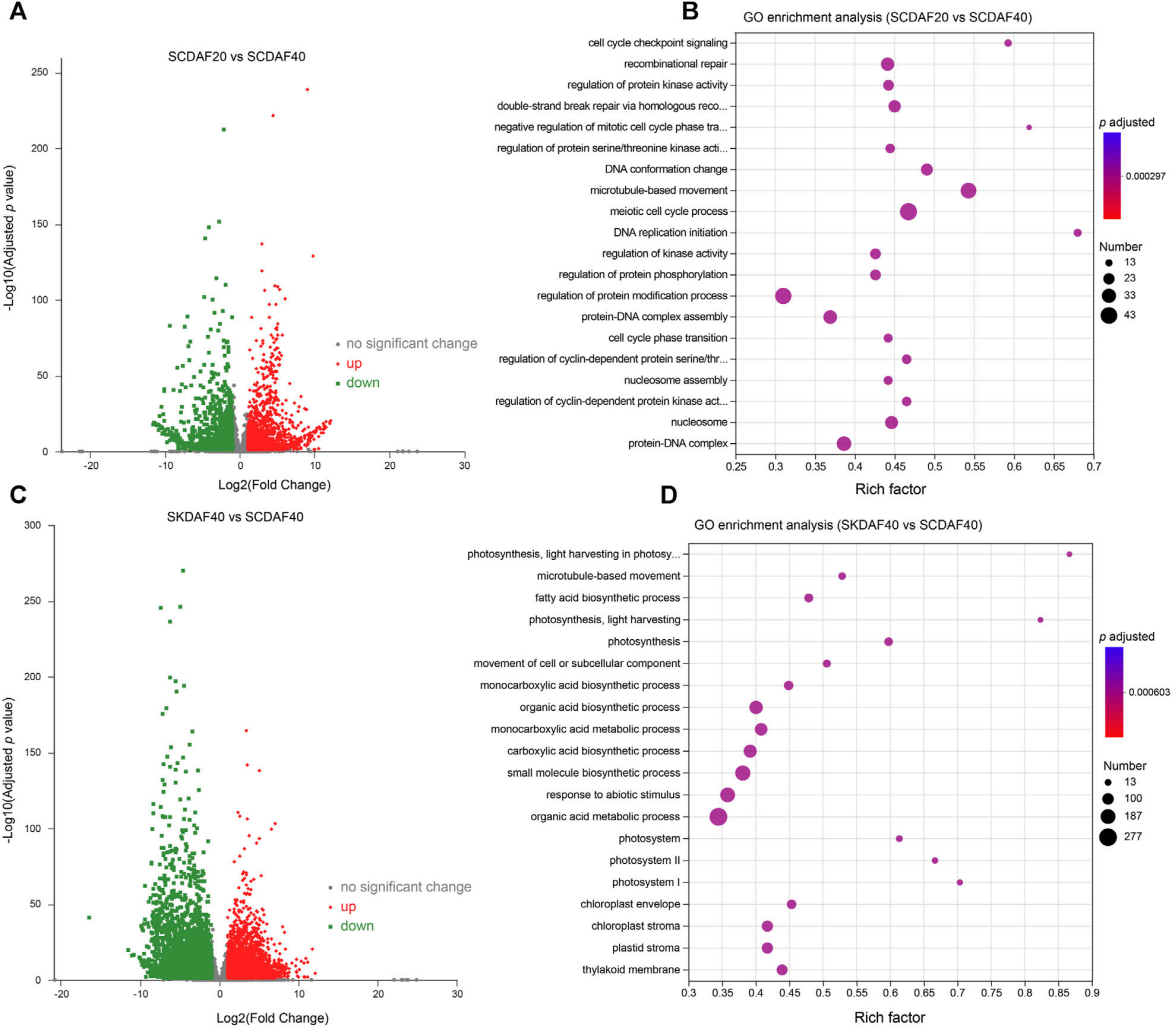
Differentially expressed gene (DEG) analysis of the comparison groups SCDAF20 vs. SCDAF40 and SKDAF 40 vs. SCDAF40. **(A, C)** Volcano maps of gene expression in SCDAF20 vs. SCDAF40 and SKDAF 40 vs. SCDAF40, respectively. The red color represents upregulated genes, and the green color represents downregulated genes. **(B, D)** indicate GO enrichment terms of DEGs in SCDAF20 vs. SCDAF40 and SKDAF 40 vs. SCDAF40, respectively.

To investigate the gene expression differences between two developmental stages of the seed coat and between the seed coat and the seed kernel at DAF40, differential expression analysis was performed using the criteria of log2(FC) ≥ 2 and FDR ≤0.05 for the comparisons of SCDAF20 vs. SCDAF40 and SCDAF40 vs. SKDAF40. The results are presented in Figure 4. In the SCDAF20 vs. SCDAF40 comparison, 3650 DEGs were identified, including 2073 upregulated and 1577 downregulated genes (Figure 4A). The GO enrichment analysis showed that the pathways in which the differentially expressed genes were primarily enriched were pathways such as microtubule-based movement, recombination repair, regulation of protein modification, and protein-DNA complex (Figure 4B). In the SCDAF40 vs. SKDAF40 comparison, 6788 differentially expressed genes were identified, including 3276 upregulated and 3512 downregulated genes (Figure 4C). The GO enrichment analysis showed that the pathways in which the differentially expressed genes were primarily enriched were organic acid and monocarboxylic acid metabolic processes, responses to abiotic stimuli, and biosynthetic processes of carboxylic acids, organic acids, and small molecules (Figure 4D). Additionally, the KEGG pathway analysis revealed that plant hormone signal transduction and flavonoid and fatty acid biosynthesis were enriched (Supplementary Figure S3A), indicating different processes of substance synthesis in the seed coat and seed kernel.

### 3.5 Gene expression time-course analysis of seed kernel development

**FIGURE 5:**
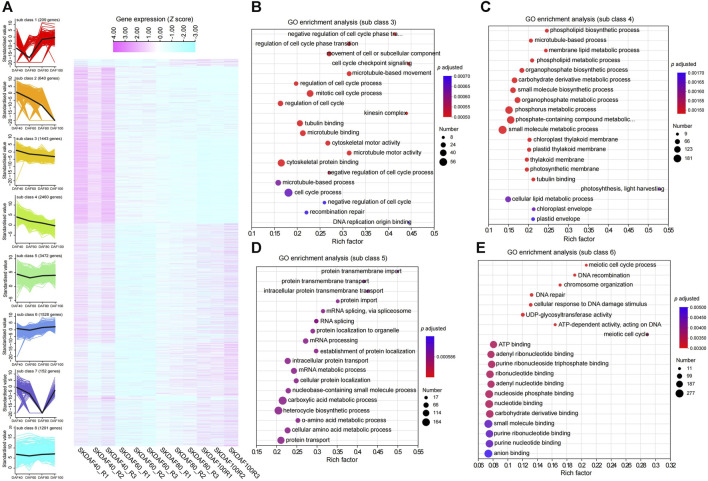
Expression profiles of specific gene classes during the development of fruits. **(A)** A total of three comparisons with 11,195 nonredundant differentially expressed genes (DEGs) were identified by RNA-seq analysis for the indicated time points. The gene expression quantification was converted to *z* scores and displayed as a heatmap according to the subclass. Higher expression levels are represented in pale violet red, while low expression levels are represented in light blue. A total of eight subclasses were obtained using k-means clustering. **(B–E)** Represent GO enrichment analysis of genes in subclasses 3, 4, 5 and 6, respectively.

To better understand the gene expression shifts during seed kernel development, we conducted differential expression analysis for three comparison groups: SKDAF40 vs. SKDAF60, SKDAF60 vs. SKDAF80, and SKDAF80 vs. SKDAF100. We identified 5310, 4749, and 6039 differentially expressed genes (DEGs), respectively, including 662 shared DEGs and 2264, 2101, and 2589 specifically expressed DEGs in the three corresponding comparison groups (Supplementary Figure S3B). *K*-means clustering was further used to classify all DEGs into eight subclasses, with subclasses 1 to 8 containing 209, 640, 1443, 2460, 3472, 1528, 152, and 1291 DEGs, respectively (Figure 5A; Supplementary Table S4). The overall expression levels of DEGs in Cluster 4 were the highest among all clusters. According to the eight sub-classes, we found that some DEGs were abundant in specific stages. For example, sub-class 1 was mainly expressed in SKDAF80 and SKDAF100, subclass 2 was mainly expressed in SKDAF40 and SKDAF60, and subclass 7 was expressed in SKDAF40, SKDAF60, and SKDAF100. There were also subclasses with high expression abundance in all stages, such as subclasses 3, 4, 5, 6, and 8. The results showed that these DEGs were characterized by complex expression patterns and might be relevant to the formation of yellowhorn fruit. The GO analyses revealed that the pathways in which the genes were enriched were mainly endopeptidase activity for subclass 1, extracellular substances for subclass 2, cell cycle-related processes for subclass 3, metabolic biosynthetic pathways or processes for subclass 4, mRNA or amino acid (protein) metabolic pathways for subclass 5, DNA and energy metabolism for subclass 6 and protein-related biosynthesis, folding and assembly for subclass 8 (Figures 5B–E; Supplementary Figures S4A–C). No GO term was observed for subclass 7. In particular, fatty acid biosynthesis was found by KEGG analysis to be enriched in subclass 8, with the genes including EVM0012846, EVM0012231, EVM0023103, EVM0024450, EVM0015606, EVM0016622, EVM0005377, EVM0007020, and EVM0007140 (Supplementary Figure S4D).

### 3.6 Weighted gene coexpression network analysis (WGCNA)

**FIGURE 6:**
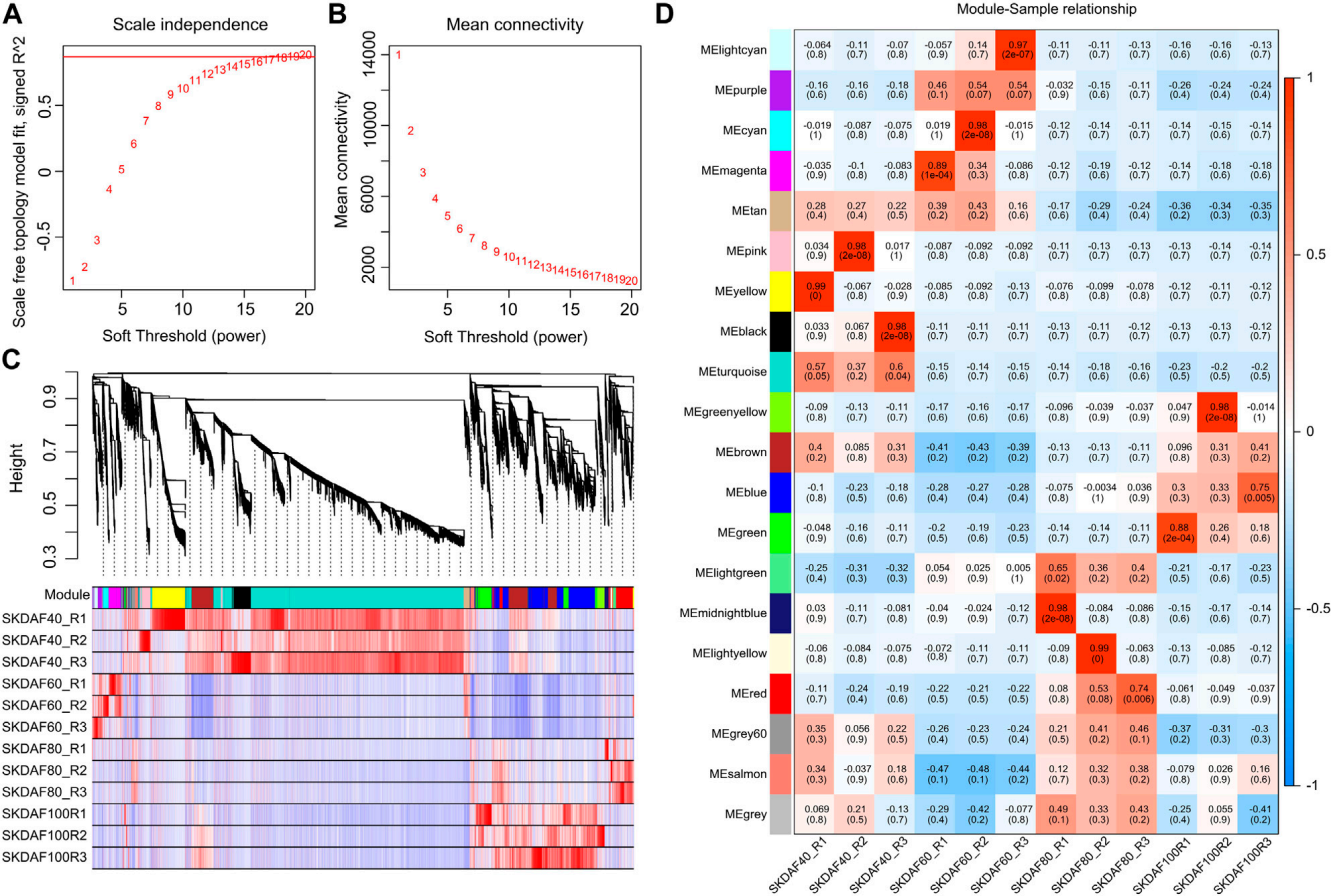
Coexpression of genes expressed in SKDAF40, SKDAF6, 0SKDAF80 and SKDAF100 by WGCNA. **(A,B)** represent scale independence and mean connectivity to determine the soft threshold (β value) at which the gene coexpression network became close to scale-free. **(C)** Co-expression module detection by hierarchical cluster tree. Genes with correlation >0. Seventy-five were merged into one module. The different colors represent the corresponding modules. **(D)** Heatmap of correlations between the four stage samples and the detected modules. The color scale on the right side shows module-trait correlation from −1 (blue) to 1 (red).

Weighted gene coexpression network analysis (WGCNA) was used to mine tissue-specific modules and key genes related to the phenotype. To explore key genes and coexpression networks that play important roles during the development of fruit, we conducted WGCNA based on the gene expression dataset of SKDAF40, SKDAF60, SKDAF80, and SKDAF100 (TPM>1). The weight value was calculated using the function pick soft threshold in the WGCNA package, and the soft threshold *β* = 18 was determined when the fitting curve was close to 0.9 for the first time (Figures 6A, B). The modules were divided based on the dynamic cutting tree, and the small modules with high similarities were merged. Then, the modules with similar expression were merged by the dynamic cutting tree method, and a total of 23001 genes in 20 coexpressed gene modules were obtained (Figure 6C). Among them, the turquoise module had the largest number of genes (10300), while the number of genes in the gray modules was the least, both containing 33 genes (Supplementary Table S5). To explore the specific modules of seed kernels during fruit development, the correlation coefficient labeled by a heatmap was used to visualize and analyze the relationship between the modules and samples. The module with a correlation coefficient above 0.80 and *p* < 0.05 was defined as the sample-specific module, and a total of 10 tissue-specific modules were obtained in the 12 samples among 20 modules (Figure 6D). Significant and positive correlations were observed between modules of pink, yellow and black and SKDAF40; light cyan, cyan and magenta were positively correlated with SKDAF60 with extreme significance; there was a significant positive correlation between midnight blue and light yellow with SKDAF80; and green and green yellow had a strong and significant correlation with SKDAF100.

### 3.7 Construction of the fatty acid biosynthesis pathway in seed kernels

**FIGURE 7:**
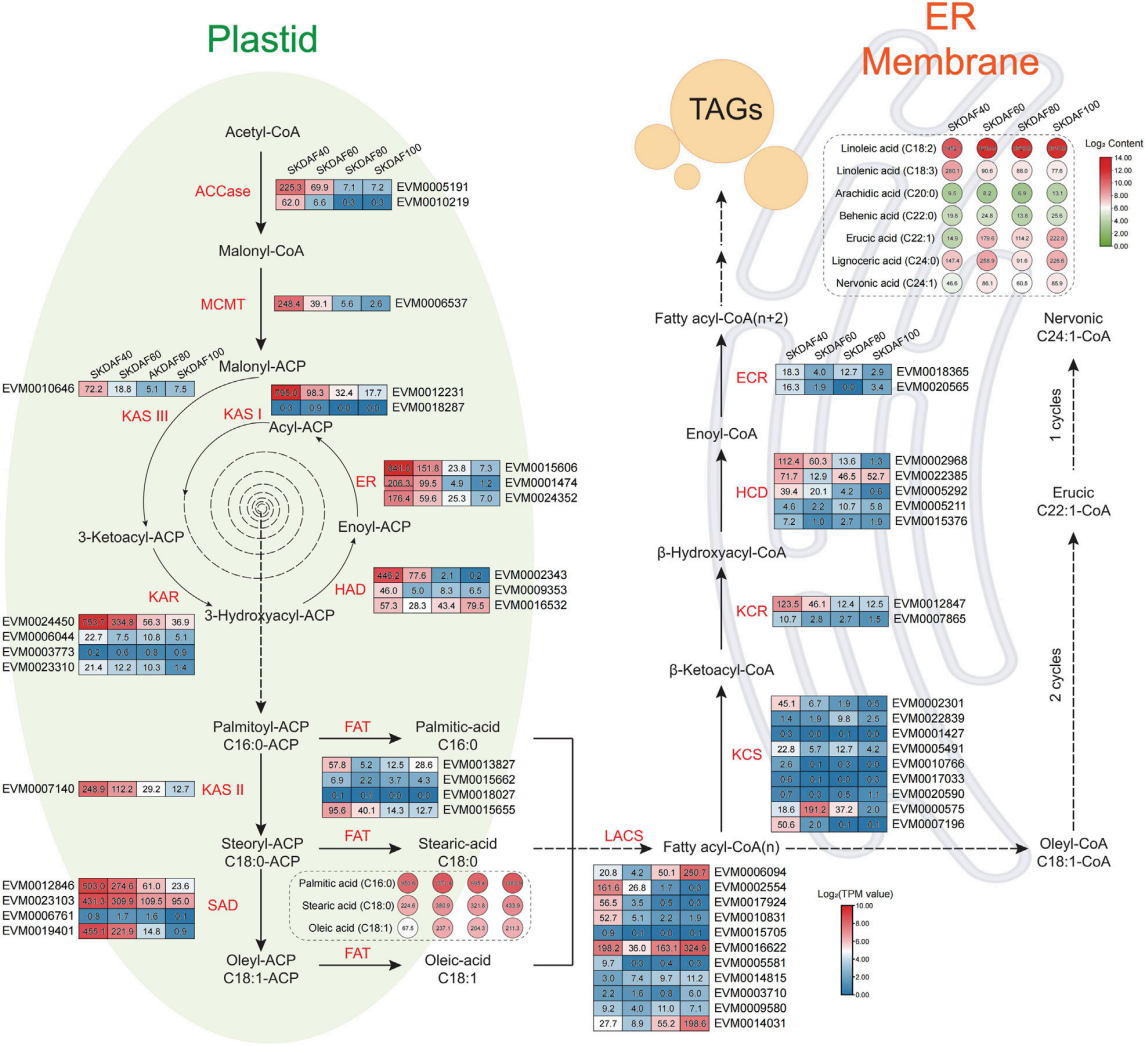
Schematic diagram of the fatty acid biosynthesis pathway in the seed kernel of yellowhorn. The rectangle indicates the expression patterns of fatty acid biosynthesis pathway-involved genes at DAF40, DAF60, DAF80 and DAF100 (from left to right of every gene). Red indicates high gene expression, and blue indicates low gene expression. The circle indicates the contents of fatty acid-related metabolites at DAF40, DAF60, DAF80 and DAF100 (from left to right of every metabolite). The red color indicates high metabolite content, and the green color indicates low metabolite content.

Fatty acids are the most economically valuable metabolites in the fruits of yellowhorn, and understanding the fatty acid synthesis pathway is important for the development and utilization of lipids. We identified 55 genes involved in the fatty acid synthesis pathway based on the genome sequence of *X*. *sorbifolia* (Supplementary Table S6) and integrated transcriptome and fatty acid content to construct a fatty acid synthesis pathway diagram (Figure 7). In the plastid processing step, almost all fatty acid synthase genes, including ACCase (EVM0005191, EVM0010219), MCMT (EVM0006537), KAS III (EVM0010646), KAR (EVM0024450), KAR I (EVM0012231), HAD (EVM0002343, EVM0009353), ER (EVM0015606, EVM0001474, EVM0024352), KAS II (EVM0007140), SAD (EVM0012846, EVM0023103, EVM0019401), and FAT (EVM0015655), were strongly expressed in SKDAF40 and gradually decreased from stage SKDAF60 to SKDAF100. Correspondingly, in the SKDAF40 stage, palmitic acid content (C16) was higher than stearic acid content (C18) and oleic acid content (C18), followed by gradual accumulation of these three fatty acids to reach the highest level in SKDAF100. The above results indicated that fatty acid synthesis-related genes are highly expressed and synthesize C16-type fatty acids in the early stage of seed development, and C18-type fatty acids have already begun to synthesize, followed by gradual accumulation of these fatty acids until fruit ripening. The LACS genes EVM0002554, EVM0017924, and EVM0010831 were highly expressed in the SKDAF40 stage and then rapidly decreased to lower expression levels. Conversely, EVM0006094 and EVM0014031 were highly expressed in SKDAF80 cells and gradually increased in SKDAF100 cells. EVM0016622 maintained a high expression level throughout the entire developmental process. In the endoplasmic reticulum membrane reaction step, the KCS homolog EVM0007196, KCR homolog EVM0012847, HCD homolog EVM0002968 and EVM0005292 were highly expressed in the SKDAF40 stage and gradually decreased to lower levels. The KCS homolog EVM0000575 rapidly increased from low expression in SKDAF40 to a peak at SKDAF60 and then continued to decline. The HCD homolog EVM022385 showed a trend of high expression followed by a decrease and then an increase. Linoleic acid was the most abundant metabolite among all fatty acids in the endoplasmic reticulum membrane reaction stage, and arachidic acid and behenic acid were present at low levels, consistent with the fatty acid content type in yellowhorn seeds. Linoleic acid and lignoceric acid peaked at SKDAF60, while linolenic acid peaked at SKDAF40 and then decreased to a stable level.

To further investigate the regulatory role of transcription factors (TFs) in the process of fatty acid biosynthesis, we annotated all expressed TFs and their potential targets and performed correlation analysis between TFs whose targets included 55 fatty acid biosynthesis-related genes (Supplementary Figure S5). The results showed that HB-other, bHLH, and ARF had strong correlations with the majority of fatty acid biosynthesis-related genes such as KCS (EVM0017033) and LACS (EVM0017924). In addition, the correlation networks between TFs and fatty acid metabolites were analyzed according to Pearson’s correlation coefficient (*p <* 0.01) and TFs like EVM0014413, EVM0023971, EVM0007638, EVM0005567 and EVM0005055 were remarkably positive or negative correlated with metabolites like linoleic acid, cis-9-octadecenoic acid, nervonic acid and decanoic acid (Supplementary Figure S6), suggesting the regulatory role of these TFs in the biosynthesis of fatty acids.

### 3.8 Real-time quantitative PCR (RT-qPCR) confirmation of fatty acid biosynthesis pathway genes

**FIGURE 8:**
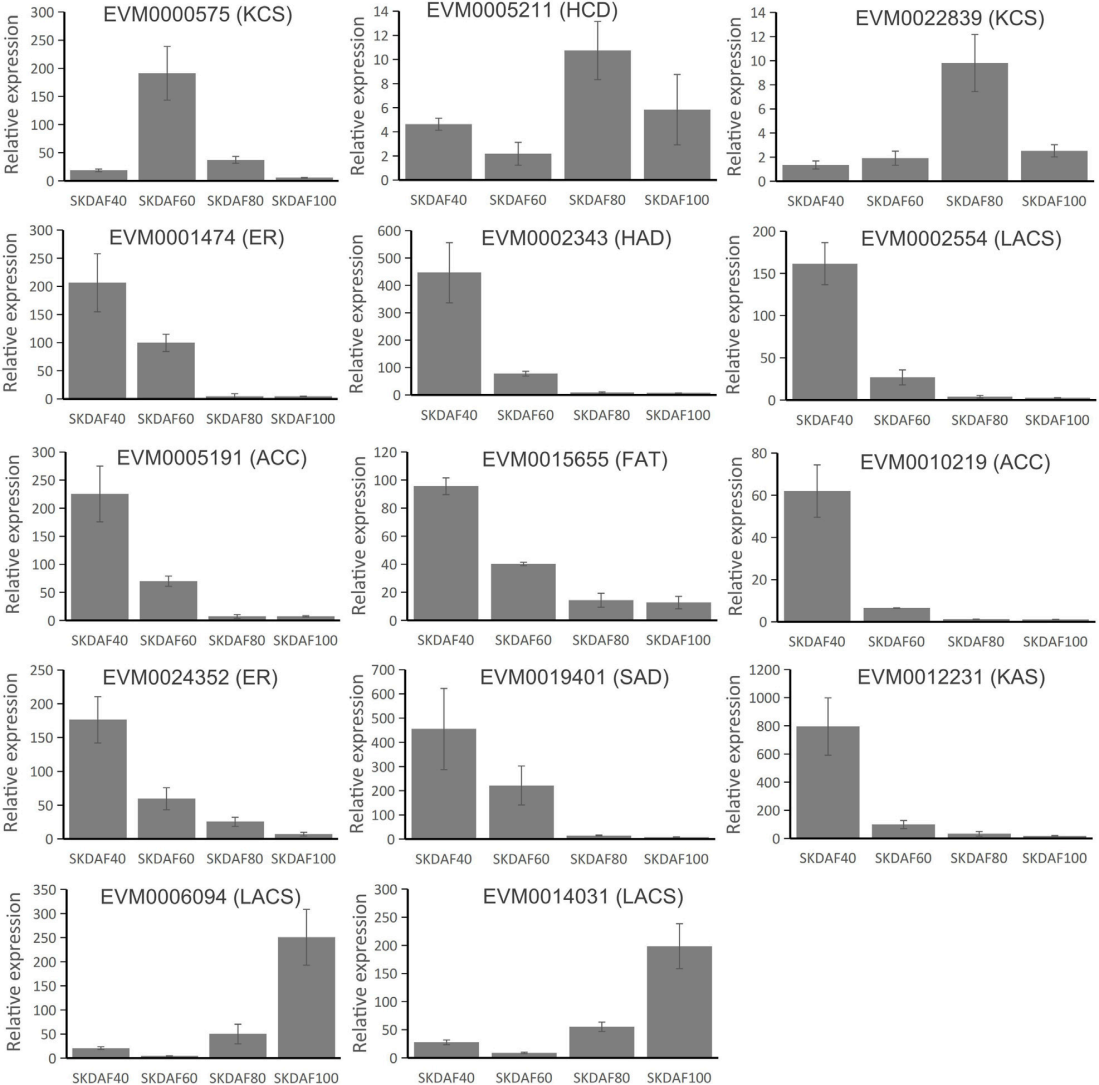
Real-time quantitative PCR of 14 fatty acid biosynthesis pathway genes. The corresponding fatty acid biosynthesis pathway gene names are labeled above the histogram. Standard deviations are shown with error bars.

To confirm the expression pattern of fatty acid biosynthesis pathway genes during the development of fruits in yellowhorn, a total of 14 genes were used for RT-qPCR in these samples (Figure 8). EVM0000575 (KCS), EVM0005211 (HCD) and EVM0022839 (KCS) showed obviously higher expression in the mid-process of development, although at different time points. Most of the selected genes, including EVM0001474 (ER), EVM0002343 (HAD), EVM0002554 (LACS), EVM0005191 (ACC), EVM0015655 (FAT), EVM0010219 (ACC), EVM0024352 (ER), EVM0019401 (SAD) and EVM0012231 (KAS), were expressed in a continuing downward trend from SKDAF40 to SKDAF100. The expression profiles of EVM0006094 (LACS) and EVM0014031 (LACS) were relatively similar, being significantly highly expressed at SKDAF100 and weakly expressed at SKDAF60. Most of the expressed genes showed similar expression trends with RNA-seq data.

## 4 Discussion

During development, seeds undergo cell division and an increase in cell volume, resulting in dynamic changes in external morphology and physical traits, such as seed size, volume, fresh and dry weight, seed coat color, and metabolites. The most significant change during development is seed size (Kesavan et al., 2013), with increased seed size and volume (Guo et al., 2009). Understanding the development of plant fruit is crucial to gaining insights into plant growth patterns and fruit quality formation, improving fruit yields, and assisting in fruit orchard management. Most plants follow an “S”-shaped curve in the changes of various traits during the entire growth period of their fruits (Wan et al., 2011). This article focuses on the study of the development of *X*. *sorbifolia* fruit (lasting approximately 100 days), which can be divided into five periods to identify the morphological and intracellular changes in the fruit samples (Figure 1). The fruits, seeds, and kernels of *X*. *sorbifolia* all undergo significant changes during development, including length and width, fresh weight, and fruit skin thickness. The first stage is from DAF 0 to 20, during which the growth is slow. The second stage is from DAF 20 to 60, where the fruit, seed, and kernel grow rapidly and reach their maximum size, with length and width reaching their peak values at this stage. It is speculated that this is the rapid cell division period of the fruit. Therefore, ensuring timely irrigation and sufficient fertilizer supply and sunlight during cultivation are important for the healthy development of *X*. *sorbifolia* seeds. After this period, as the fruit gradually matures and loses moisture, the fresh weight of the fruit changes significantly due to its larger size compared to the seeds and kernel. Analysis of fruit vertical diameter and transverse diameter showed that the vertical growth of the fruit was faster than the horizontal growth before DAF40, but the horizontal growth became faster after DAF40, indicating that independent genes may control vertical and horizontal growth of the fruit. The main nutrients in *X*. *sorbifolia* are lipids, soluble sugars, proteins, etc., which are stored nutrients from photosynthesis in the plant. The accumulation of starch, sugar, and lipids varies in different plant seeds, but there is a close relationship between them (Hapeta et al., 2017; Xu et al., 2019). The soluble sugar content in the seed coat significantly increases, while the starch content does not change significantly, indicating that soluble sugar may be synthesized in the seed coat and transported to the kernel. As the seed matures, the seed coat flesh gradually hardens, and further analysis of the seed coat during its entire development period is needed to determine its metabolic component changes. From DAF40 to DAF80, the soluble sugar, starch, and anthocyanin content in the kernel showed an increasing trend, but they decreased significantly at DAF100. This may be due to soluble sugar and starch gradually converting into lipids in the kernel as the fruit matures. Therefore, there is an overall trend of increasing lipid content and decreasing soluble sugar and protein content. Soluble protein content stops increasing as seeds mature, indicating that proteins in different periods are generally the same but may have some differences in their types and contents. Future research could focus on studying protein expression in different periods to understand their differences and specific functions.

During the development of yellowhorn fruit, the seed coat may have an impact on the development of seed kernels. However, there have been limited reports on transcriptional regulation in the seed coat of yellowhorn. In this study, transcriptional regulation of DAF20 and DAF40 seed coat samples was compared by RNA-seq, and differential expression analysis was conducted. The DEGs between SCDAF20 vs. SCDAF40 were mainly enriched in microtubule-based movement, recombination repair, regulation of protein modification, and protein-DNA complex (Figure 4B), indicating their strong vital movement. In the SCDAF40 vs. SKDAF40 comparison, 6788 differentially expressed genes were identified, enriched in organic acid and monocarboxylic acid metabolic processes, responses to abiotic stimuli, and biosynthetic processes of carboxylic acids, organic acids, and small molecules (Figure 4D), indicating that transcriptional differentiation between the seed coat and kernel is significant at DAF40 and that metabolites are already synthesized within the kernel.

In plants, photosynthesis synthesizes carbohydrates, which may be converted into important energy-rich substances called lipids. The fatty acid biosynthetic pathway in plants has been well characterized (He et al., 2020). In this study, we identified 54 genes related to the biosynthesis of oil in yellowhorn, including 25 genes involved in plastid synthesis and 18 genes participating in endoplasmic reticulum synthesis. These genes were expressed in the developing seed during different developmental stages. Through hierarchical clustering analysis, nine genes in subclass 8 were enriched in the fatty acid biosynthesis pathway. The key genes of this study involved in the *de novo* biosynthesis of fatty acids showed similar expression patterns. ACCase, MCMT, KAS, ERF, SAD, KCR, and HCD were highly expressed in DAF40 and DAF60, and their expression levels rapidly decreased afterward (Figure 7). This period corresponds to the seed development stage where the color changes from white to brown-red (Figure 1), which is a period of rapid oil accumulation. The high expression of these genes at these two stages is synchronized with rapid oil accumulation during this period, indicating that they may jointly participate in early rapid oil accumulation, which is similar to studies on pecans, tea seeds, and Artemisia sphaerocephala (Huang et al., 2017; Lin et al., 2018; Nan et al., 2021). In fatty acid synthesis, ACCase catalyzes the carboxylation of acetyl-CoA to malonyl-CoA, which is the rate-limiting step in plant fatty acid synthesis (Cui et al., 2017). In this study, we found that the ACCase gene (EVM0005191 and EVM0010219) was highly expressed at the early stage of seed development (DAF40), and its expression gradually decreased as the seed developed to lower expression levels, which was confirmed by RT-qPCR (Figure 8). A similar trend was observed in pecans (Huang et al., 2017). This result can provide a rich substrate for subsequent fatty acid synthesis. KAS initiates the elongation of fatty acid carbon chains, and C18 is primarily catalyzed by KASII. Analysis of gene expression levels found that KASI and KASII were expressed at significantly higher levels than KASIII in different stages of seed development in yellowhorn, consistent with the findings for pecan (Huang et al., 2017). Another key enzyme is SAD, which catalyzes C18:0 to C18:1 and determines the ratio of SAFs to MUFAs (Han et al., 2017). The present study identified four SAD genes, three of which were highly expressed, consistent with the high oleic acid content in seed kernels. Similar results have been reported in olive trees (Parvini et al., 2016). In conclusion, these consistently expressed genes are candidate genes for fatty acid biosynthesis and could serve as targets for future genetic improvement of yellowhorn.

The metabolism of lipids is regulated by transcription factors (Baud and Lepiniec, 2009; He et al., 2020), such as MYB (Li et al., 2017) and AP2 (Kong and Ma, 2018). Related transcription factors have also been identified in yellowhorn (Liu et al., 2013). However, the number of TFs identified to regulate fatty acid biosynthesis in yellowhorn is relatively lacking. Moreover, the relationships among these TFs regulating lipid accumulation have not been extensively explored. The transcriptome sequencing data in this study provide a chance to discern regulation and to screen new regulators involved in the lipid accumulation pathway. We identified that HB-other, bHLH, and ARF had strong positive correlations with fatty acid biosynthesis pathway genes such as KCS (EVM0017033) and LACS (EVM0017924) (*p* < 0.01) in this paper. Recent studies have revealed that HB-other, AP2, ARF, ERF and bHLH are related to lipid metabolism (Jia et al., 2018; Thiriet-Rupert et al., 2018; Kang et al., 2019; Feng et al., 2020). These TFs with high expression pattern correlations could be regarded as potential targets for genetic modification, laying a foundation for future research on the transcriptional regulation of lipid metabolism processes in yellowhorn.

## 5 Conclusion

To reveal the dynamic development of yellowhorn fruit, this study determined agronomic traits and physiological indices to characterize fruit and seed growth in five key stages. The results showed that a “slow-fast-slow” growth pattern was observed throughout the fruit developmental period. Omics data analysis indicates that there are different metabolite expressions and accumulations in the seed coat and seed kernel, and the metabolites accumulated in the seed coat may be transferred to the kernel over time. There is a gene expression shift phenomenon in the DEGs related to the development of the seed kernel, and *k*-means clustering analysis identified a subclass that may be related to fatty acid metabolism. WGCNA also identified expression modules that are highly correlated with seed kernel development. The roles of TFs in the fatty acid biosynthesis process were explored, and some potential TFs associated with fatty acid-related genes were screened. The results might lay a foundation for future regulatory mechanisms elucidating fatty acid biosynthesis and high lipid molecular breeding of yellowhorn.

## Data Availability

The datasets presented in this study can be found in online repositories. The names of the repository/repositories and accession number(s) can be found in the article/Supplementary Material.
