# Supplementary material for: Agronomic, physiological and transcriptional characteristics provide insights into fatty acid biosynthesis in yellowhorn (Xanthoceras sorbifolium Bunge) during fruit ripening
Source: Front Genet. 2024 Jan 31;15:1325484. doi: 10.3389/fgene.2024.1325484 (PMC10864670; doi:10.3389/fgene.2024.1325484)
Supplement: Supplementary file 1 [file DataSheet1.ZIP › supplementary figures and tables/Figure S3.pdf]

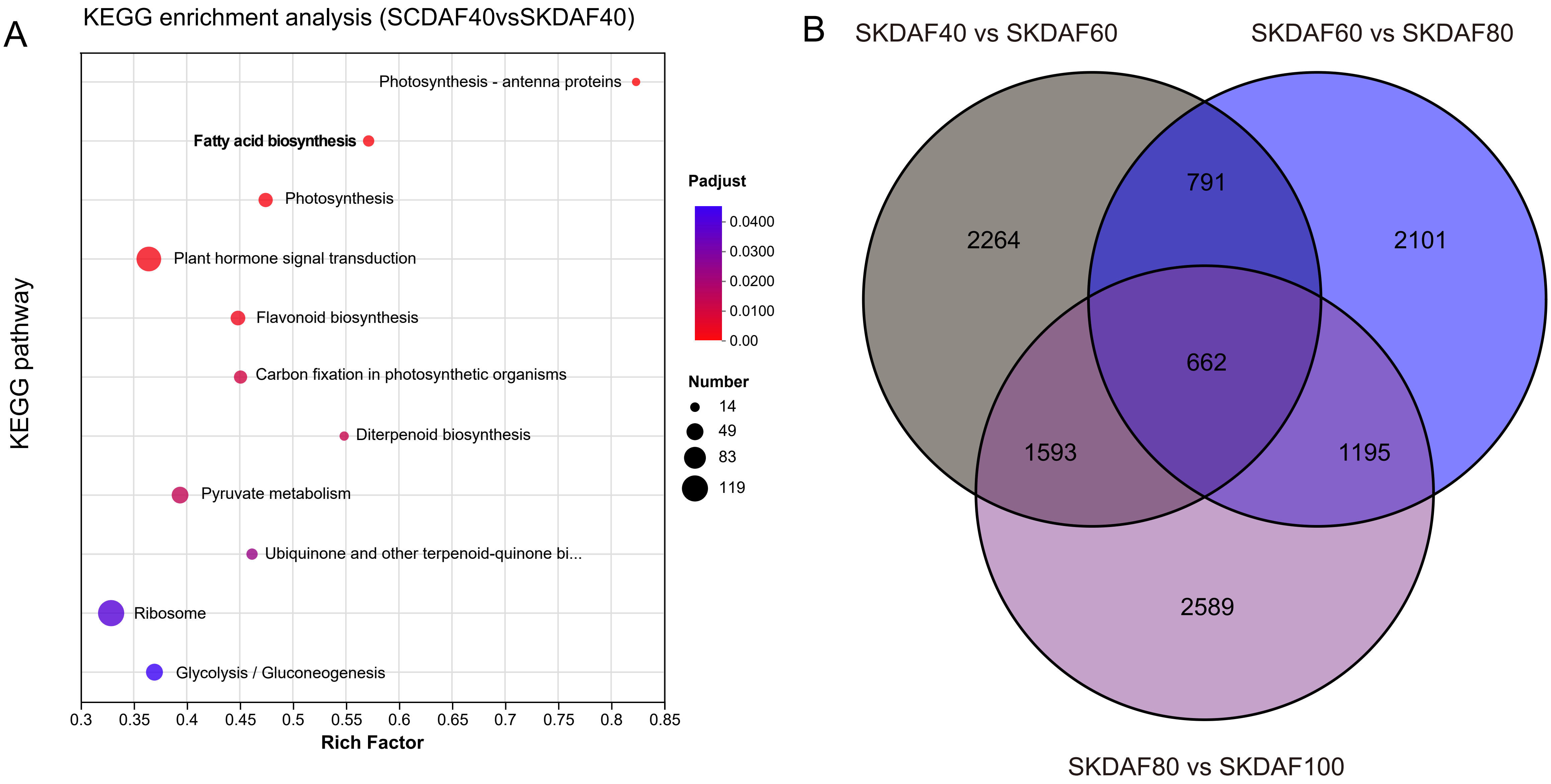
A
KEGG enrichment analysis (SCDAF40vsSKDAF40)
Photosynthesis - antenna proteins
Fatty acid biosynthesis
Photosynthesis
Plant hormone signal transduction
Flavonoid biosynthesis
Carbon fixation in photosynthetic organisms
Diterpenoid biosynthesis
Pyruvate metabolism
Ubiquinone and other terpenoid-quinone bi...
Ribosome
Glycolysis / Gluconeogenesis
KEGG pathway
0.3 0.35 0.4 0.45 0.5 0.55 0.6 0.65 0.7 0.75 0.8 0.85
Rich Factor
Padjust
0.0400
0.0300
0.0200
0.0100
0.00
Number
14
49
83
119
B
SKDAF40 vs SKDAF60
SKDAF60 vs SKDAF80
SKDAF80 vs SKDAF100
2264
791
2101
662
1593
1195
2589
