# Supplementary material for: Agronomic, physiological and transcriptional characteristics provide insights into fatty acid biosynthesis in yellowhorn (Xanthoceras sorbifolium Bunge) during fruit ripening
Source: Front Genet. 2024 Jan 31;15:1325484. doi: 10.3389/fgene.2024.1325484 (PMC10864670; doi:10.3389/fgene.2024.1325484)
Supplement: Supplementary file 1 [file DataSheet1.ZIP › supplementary figures and tables/Figure S4.pdf]

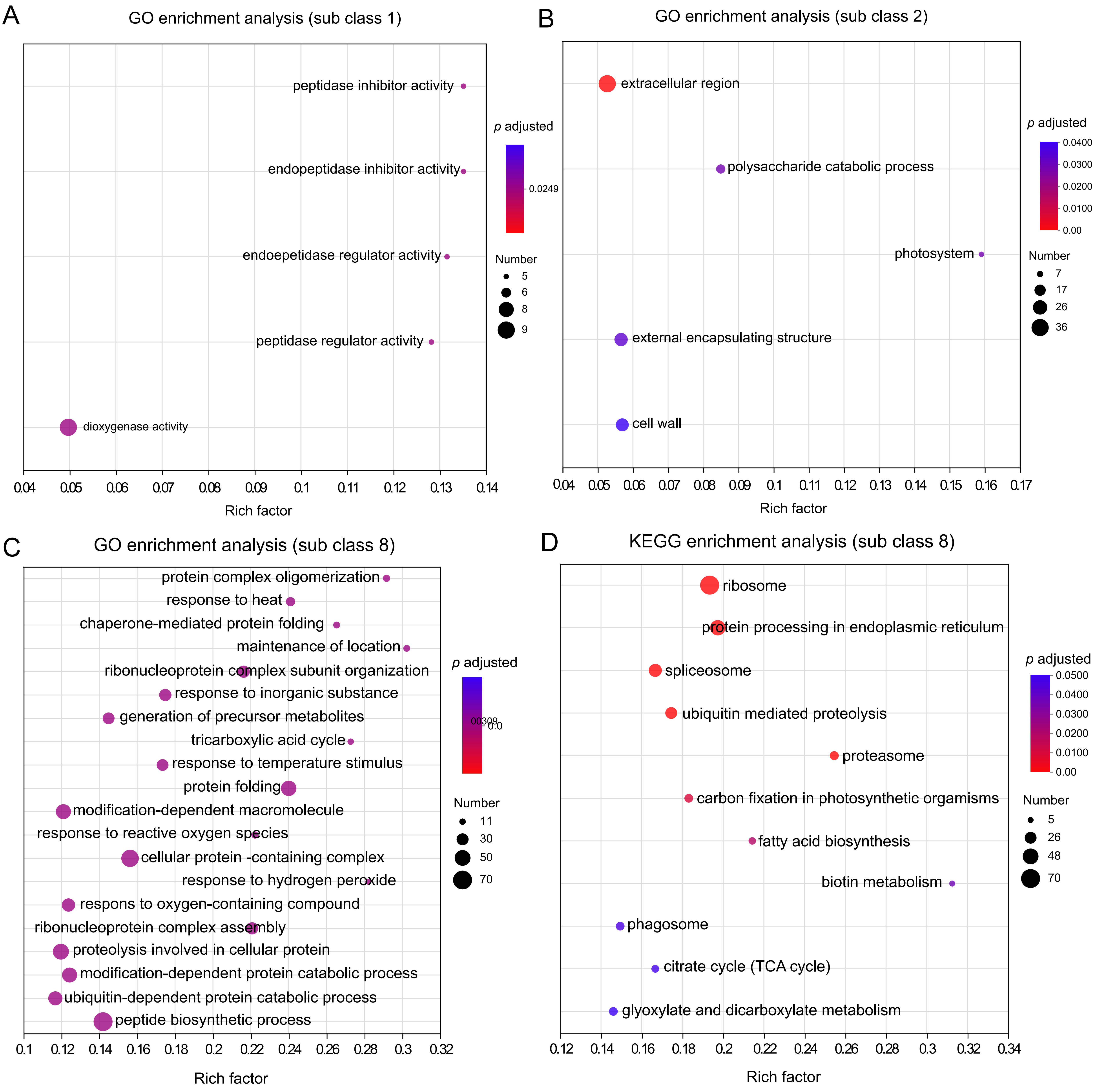
A
GO enrichment analysis (sub class 1)
peptidase inhibitor activity
endopeptidase inhibitor activity
endoepetidase regulator activity
peptidase regulator activity
dioxygenase activity
0.04 0.05 0.06 0.07 0.08 0.09 0.1 0.11 0.12 0.13 0.14
Rich factor
p adjusted
0.0249
Number
5
6
8
9
B
GO enrichment analysis (sub class 2)
extracellular region
polysaccharide catabolic process
photosystem
external encapsulating structure
cell wall
0.04 0.05 0.06 0.07 0.08 0.09 0.1 0.11 0.12 0.13 0.14 0.15 0.16 0.17
Rich factor
p adjusted
0.0400
0.0300
0.0200
0.0100
0.00
Number
7
17
26
36
C
GO enrichment analysis (sub class 8)
protein complex oligomerization
response to heat
chaperone-mediated protein folding
maintenance of location
ribonucleoprotein complex subunit organization
response to inorganic substance
generation of precursor metabolites
tricarboxylic acid cycle
response to temperature stimulus
protein folding
modification-dependent macromolecule
response to reactive oxygen species
cellular protein -containing complex
response to hydrogen peroxide
respons to oxygen-containing compound
ribonucleoprotein complex assembly
proteolysis involved in cellular protein
modification-dependent protein catabolic process
ubiquitin-dependent protein catabolic process
peptide biosynthetic process
0.1 0.12 0.14 0.16 0.18 0.2 0.22 0.24 0.26 0.28 0.3 0.32
Rich factor
p adjusted
Number
11
30
50
70
D
KEGG enrichment analysis (sub class 8)
ribosome
protein processing in endoplasmic reticulum
spliceosome
ubiquitin mediated proteolysis
proteasome
carbon fixation in photosynthetic orgamisms
fatty acid biosynthesis
biotin metabolism
phagosome
citrate cycle (TCA cycle)
glyoxylate and dicarboxylate metabolism
0.12 0.14 0.16 0.18 0.2 0.22 0.24 0.26 0.28 0.3 0.32 0.34
Rich factor
p adjusted
0.0500
0.0400
0.0300
0.0200
0.0100
0.00
Number
5
26
48
70
