# Supplementary material for: Agronomic, physiological and transcriptional characteristics provide insights into fatty acid biosynthesis in yellowhorn (Xanthoceras sorbifolium Bunge) during fruit ripening
Source: Front Genet. 2024 Jan 31;15:1325484. doi: 10.3389/fgene.2024.1325484 (PMC10864670; doi:10.3389/fgene.2024.1325484)
Supplement: Supplementary file 1 [file DataSheet1.ZIP › supplementary figures and tables/Figure S5.pdf]

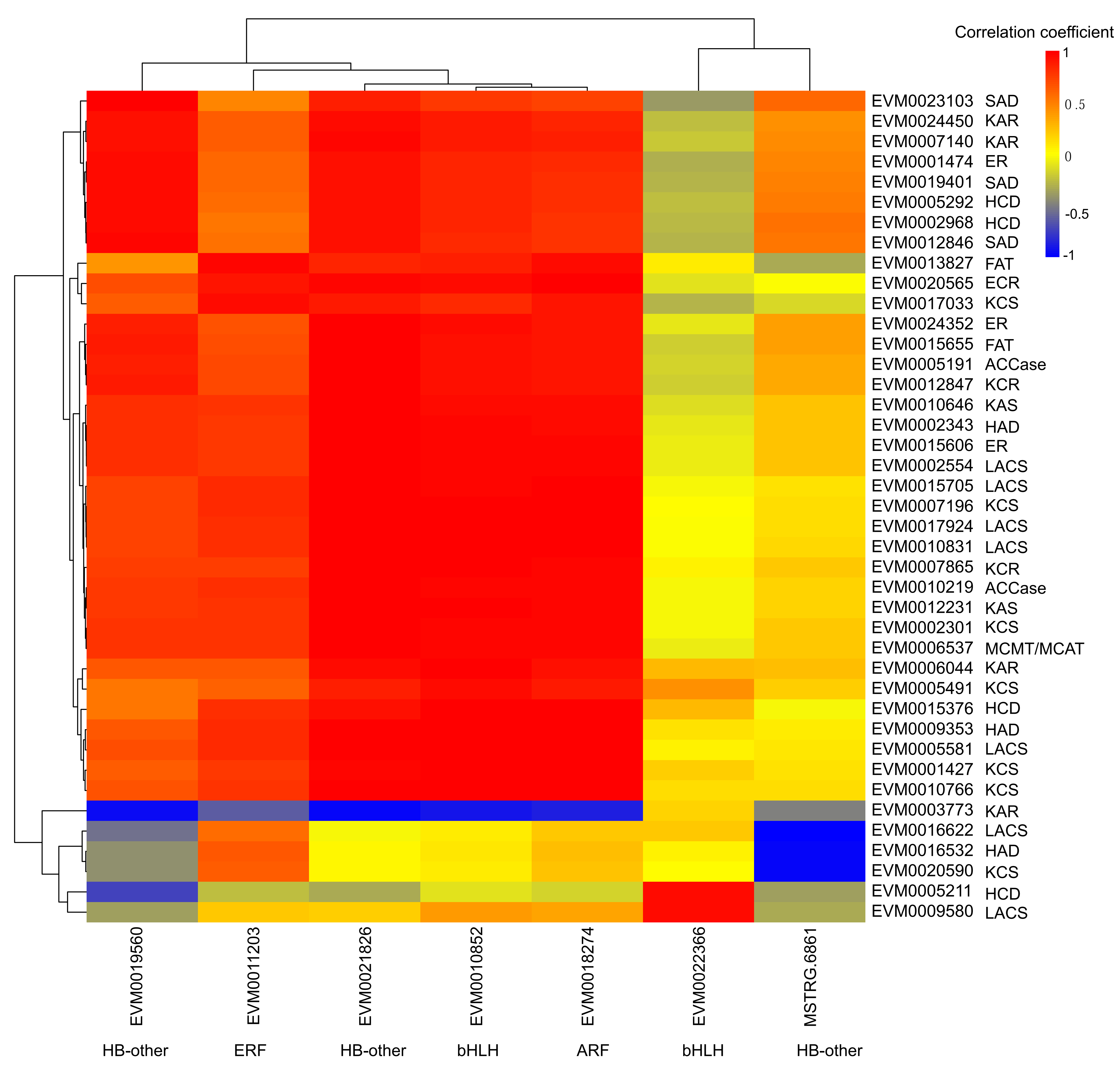

Correlation coefficient
1
0.5
0
-0.5
-1
EVM0023103 SAD
EVM0024450 KAR
EVM0007140 KAR
EVM0001474 ER
EVM0019401 SAD
EVM0005292 HCD
EVM0002968 HCD
EVM0012846 SAD
EVM0013827 FAT
EVM0020565 ECR
EVM0017033 KCS
EVM0024352 ER
EVM0015655 FAT
EVM0005191 ACCase
EVM0012847 KCR
EVM0010646 KAS
EVM0002343 HAD
EVM0015606 ER
EVM0002554 LACS
EVM0015705 LACS
EVM0007196 KCS
EVM0017924 LACS
EVM0010831 LACS
EVM0007865 KCR
EVM0010219 ACCase
EVM0012231 KAS
EVM0002301 KCS
EVM0006537 MCMT/MCAT
EVM0006044 KAR
EVM0005491 KCS
EVM0015376 HCD
EVM0009353 HAD
EVM0005581 LACS
EVM0001427 KCS
EVM0010766 KCS
EVM0003773 KAR
EVM0016622 LACS
EVM0016532 HAD
EVM0020590 KCS
EVM0005211 HCD
EVM0009580 LACS
EVM0019560 HB-other
EVM0011203 ERF
EVM0021826 HB-other
EVM0010852 bHLH
EVM0018274 ARF
EVM0022366 bHLH
MSTRG.6861 HB-other
