# Supplementary material for: Agronomic, physiological and transcriptional characteristics provide insights into fatty acid biosynthesis in yellowhorn (Xanthoceras sorbifolium Bunge) during fruit ripening
Source: Front Genet. 2024 Jan 31;15:1325484. doi: 10.3389/fgene.2024.1325484 (PMC10864670; doi:10.3389/fgene.2024.1325484)
Supplement: Supplementary file 1 [file DataSheet1.ZIP › supplementary figures and tables/Supplementary Materials.docx]

Supplementary Materials

# Supplementary Figures and Tables

## Supplementary Figures

**Supplementary Figure 1.** The appearance of yellowhorn racemes, shoots, flowers, leaves, fruits, seeds and seed kernels.

**Supplementary Figure 2.** Pearson's correlation between qRT‒PCR and RNA-seq expression quantification.

**Supplementary Figure 3.** KEGG enrichment analysis of DEGs in comparison SCDAF40 vs. SKDAF40 (A) and Venn plot of DEGs comparisons SKDAF40 vs. SKDAF60, SKDAF60 vs. SKDAF80 and SKDAF80 vs. SKDAF100 (B).

**Supplementary Figure 4.** GO and KEGG enrichment analysis of subclass genes using the k-means clustering method.

**Supplementary Figure 5.** Clustering heatmap of the correlation between the expression of TFs and fatty acid biosynthesis pathway genes. The red color and blue color indicate positive and negative correlations, respectively.

**Supplementary Figure 6.** Correlation networks between the expression of TFs and fatty acid metabolites. The networks were visualized with Cytoscape software (version 3.9.1). The square indicated fatty acid metabolites. The circle indicated TFs. The red line indicated positive correlation, while the blue line indicated negative correlation. The Pearson's correlation coefficient with r > 0.90 and *p* value *<* 0.01 were maintained.

## Supplementary Tables

**Supplementary Table 1.** Sampling times.

**Supplementary Table 2.** Primers for qRT-PCR.

**Supplementary Table 3.** RNA-seq statistics of data volume, quality and mapping rate.

**Supplementary Table 4.** Eight sub classes of DEGs using k-means cluster method.

**Supplementary Table 5.** The 20 modules of genes using WGCNA method.

**Supplementary Table 6.** Genome-wide fatty acid biosynthesis pathway genes in yellowhorn.
