# Supplementary figures and images for: Agronomic, physiological and transcriptional characteristics provide insights into fatty acid biosynthesis in yellowhorn (Xanthoceras sorbifolium Bunge) during fruit ripening
Source: Front Genet. 2024 Jan 31;15:1325484. doi: 10.3389/fgene.2024.1325484 (PMC10864670; doi:10.3389/fgene.2024.1325484)

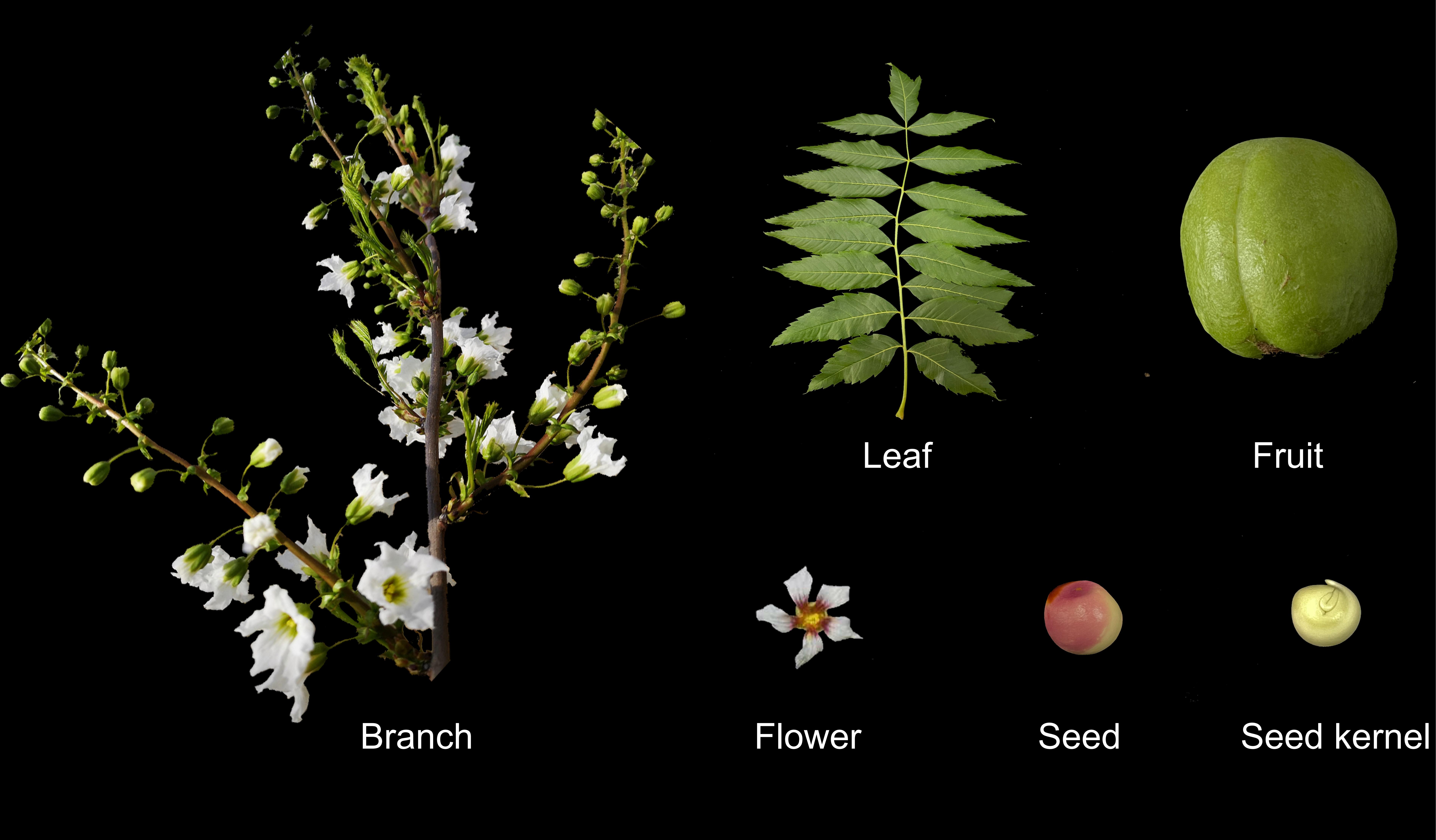

Supplement: Supplementary file 1 [file DataSheet1.ZIP › supplementary figures and tables/Figure S1.jpg]

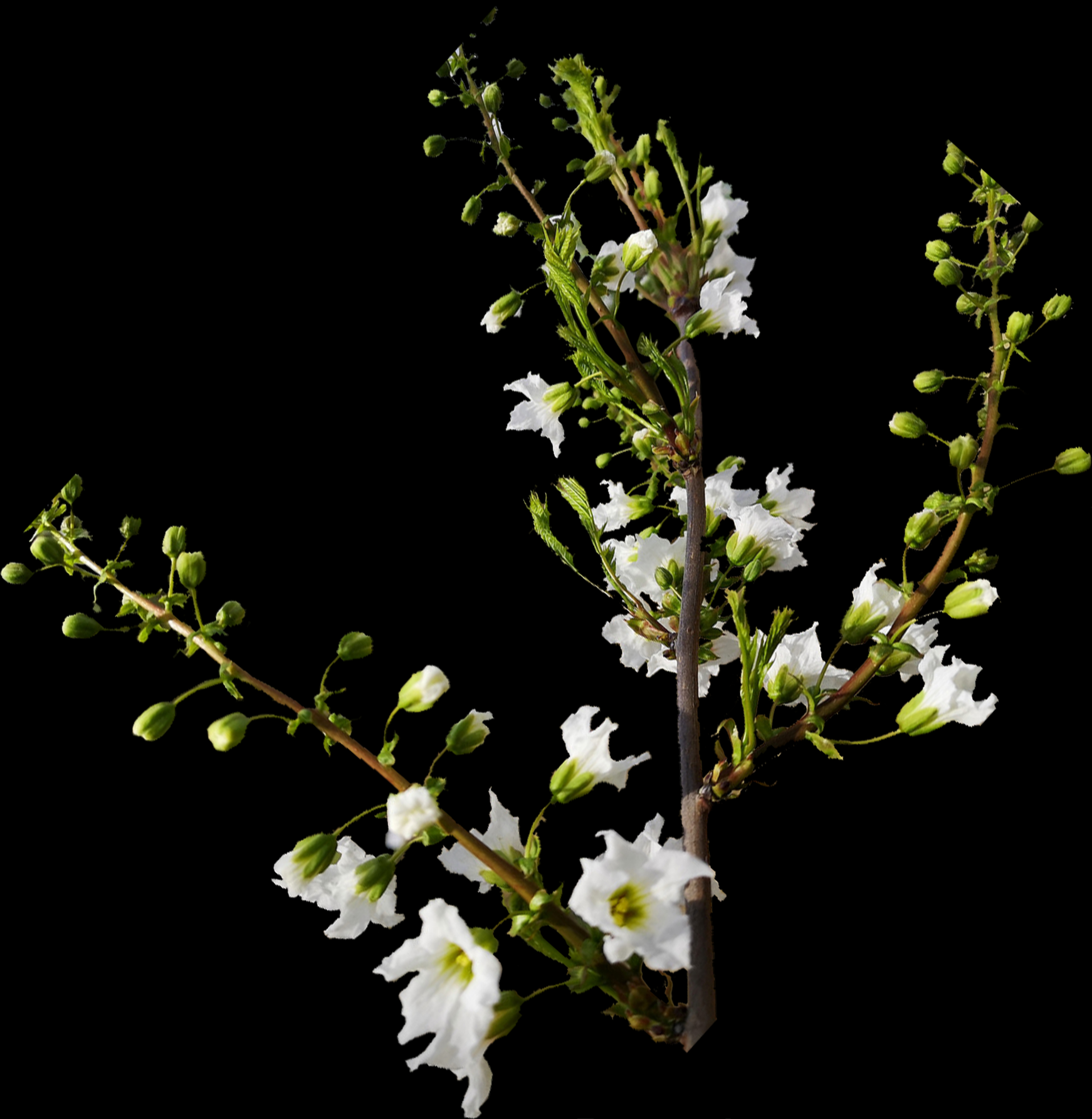

Branch

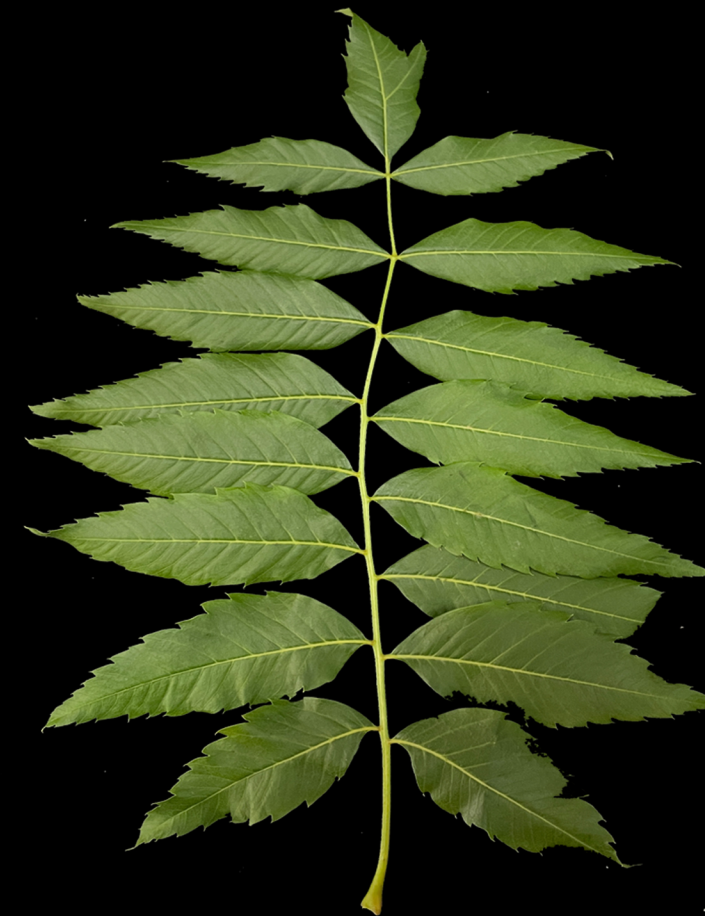

Leaf

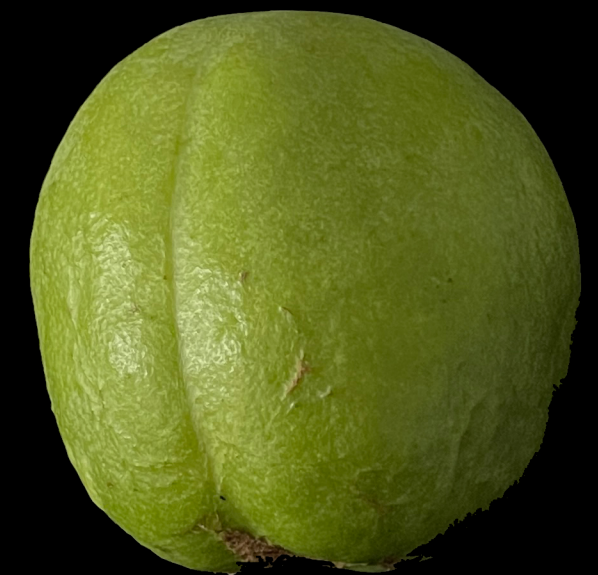

Fruit

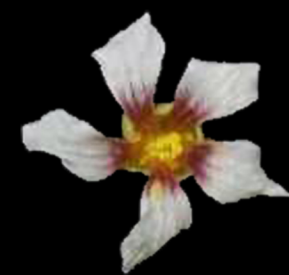

Flower

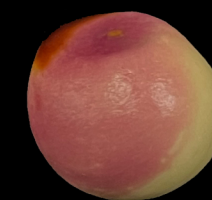

Seed

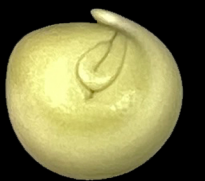

Seed kernel

Supplement: Supplementary file 1 [file DataSheet1.ZIP › supplementary figures and tables/Figure S1.pdf]

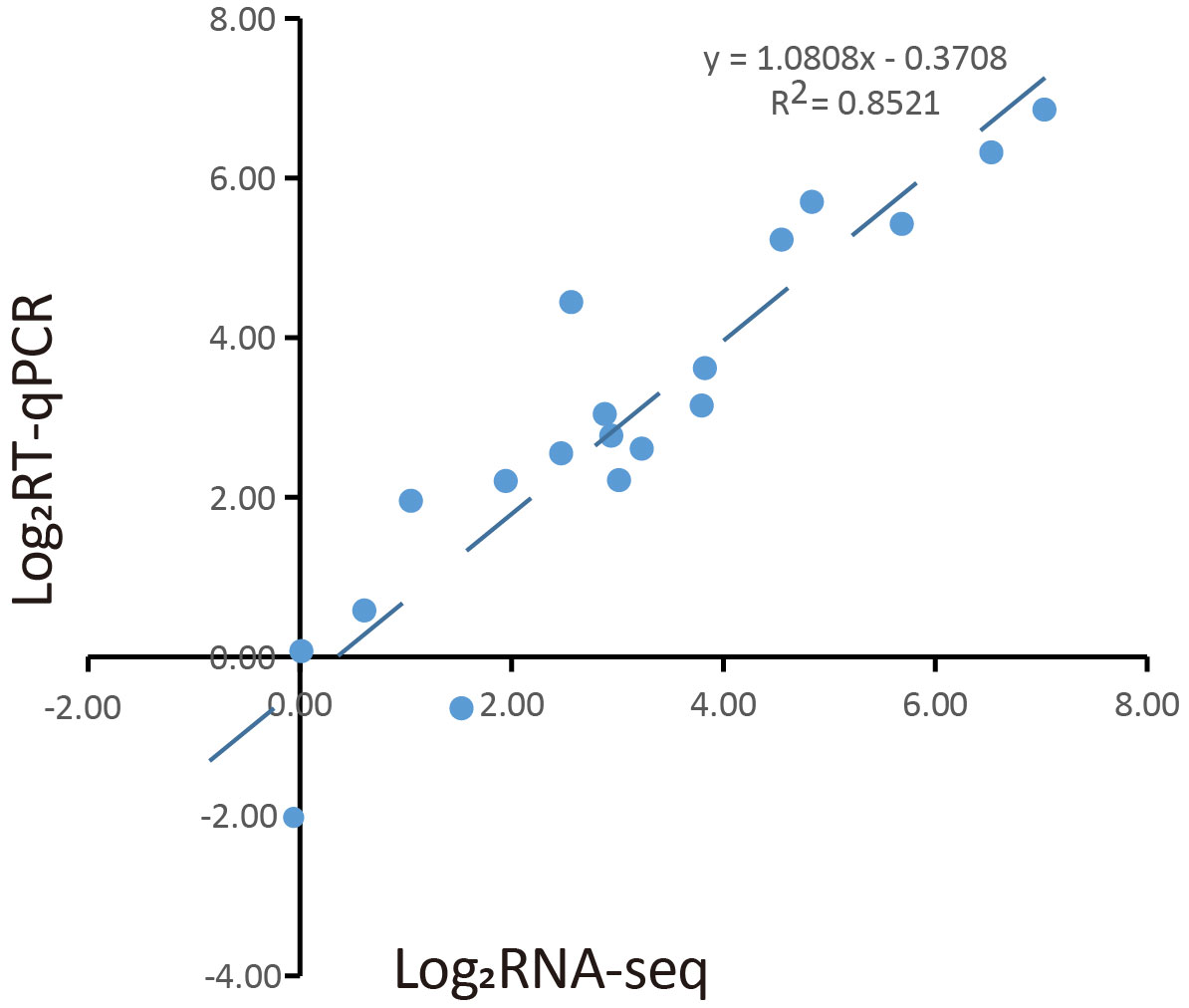

Supplement: Supplementary file 1 [file DataSheet1.ZIP › supplementary figures and tables/Figure S2.jpg]

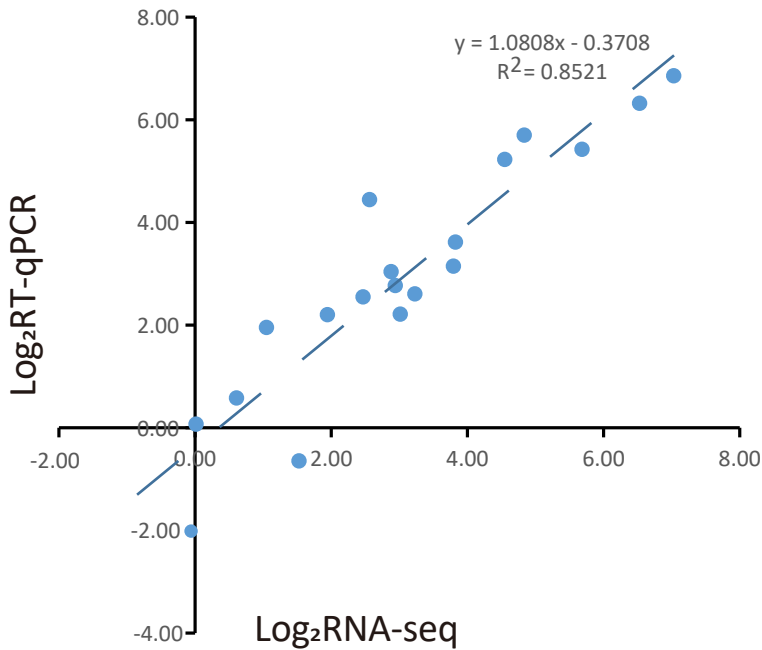

Supplement: Supplementary file 1 [file DataSheet1.ZIP › supplementary figures and tables/Figure S2.pdf]

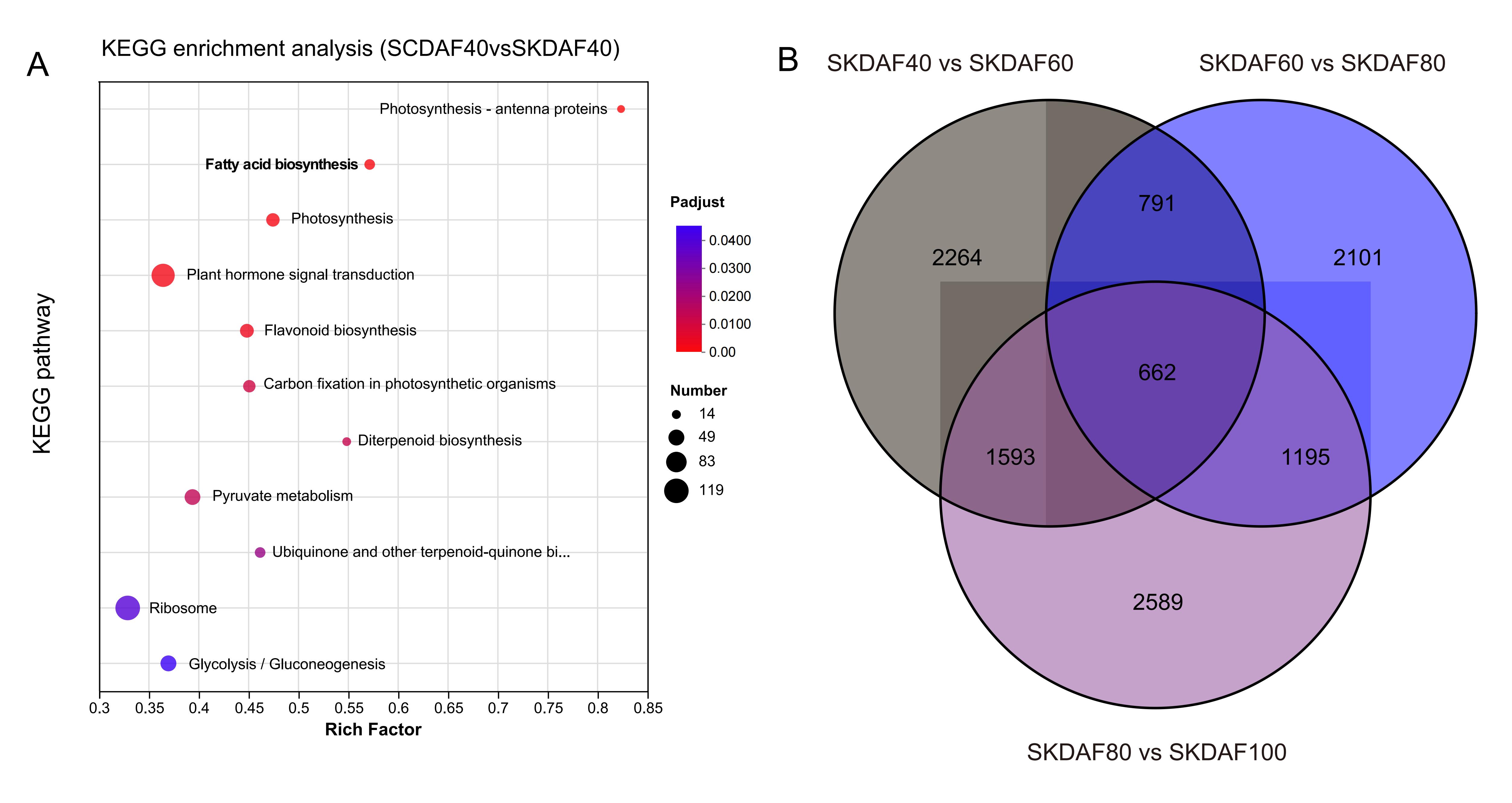

Supplement: Supplementary file 1 [file DataSheet1.ZIP › supplementary figures and tables/Figure S3.jpg]

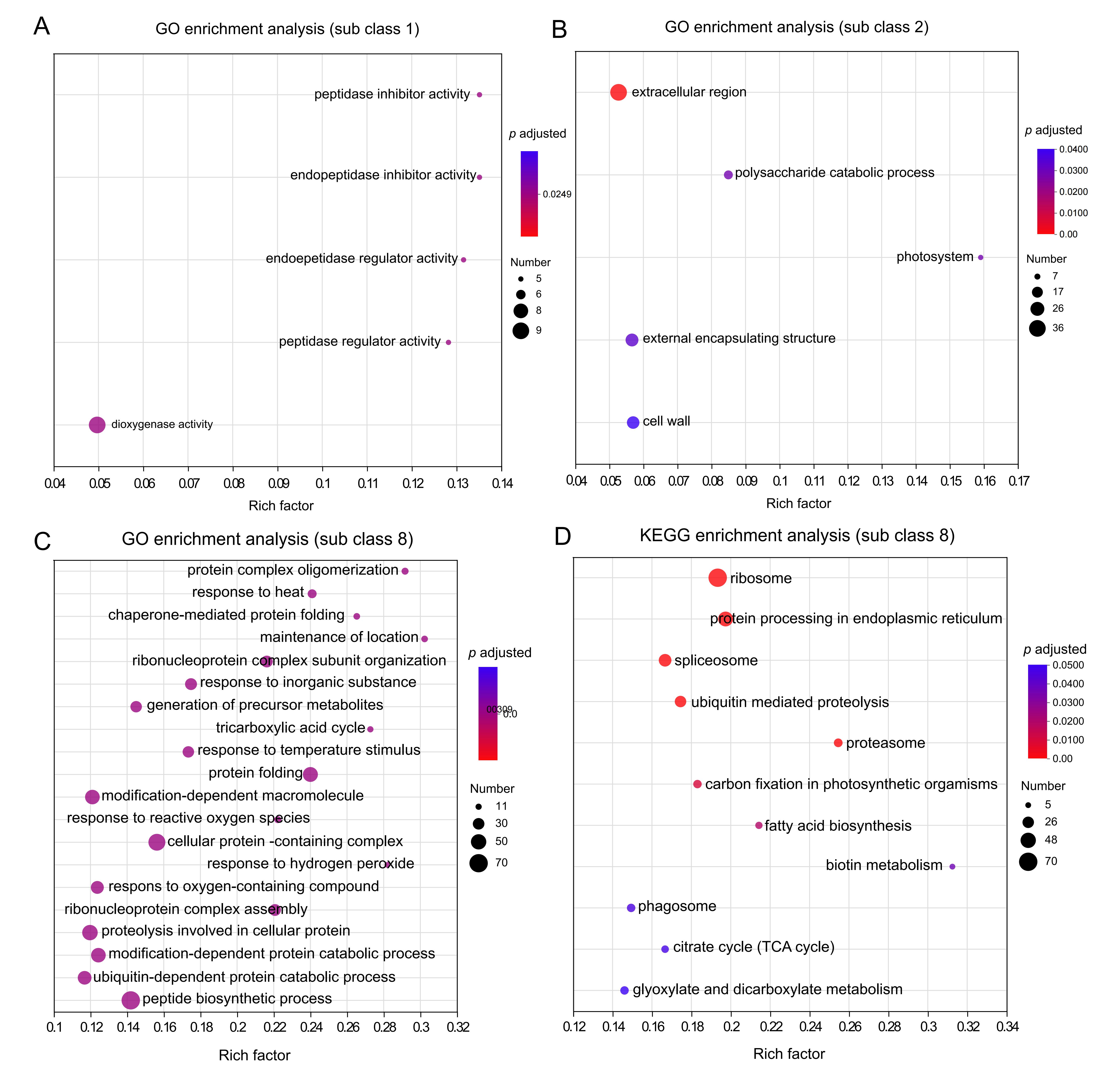

Supplement: Supplementary file 1 [file DataSheet1.ZIP › supplementary figures and tables/Figure S4.jpg]

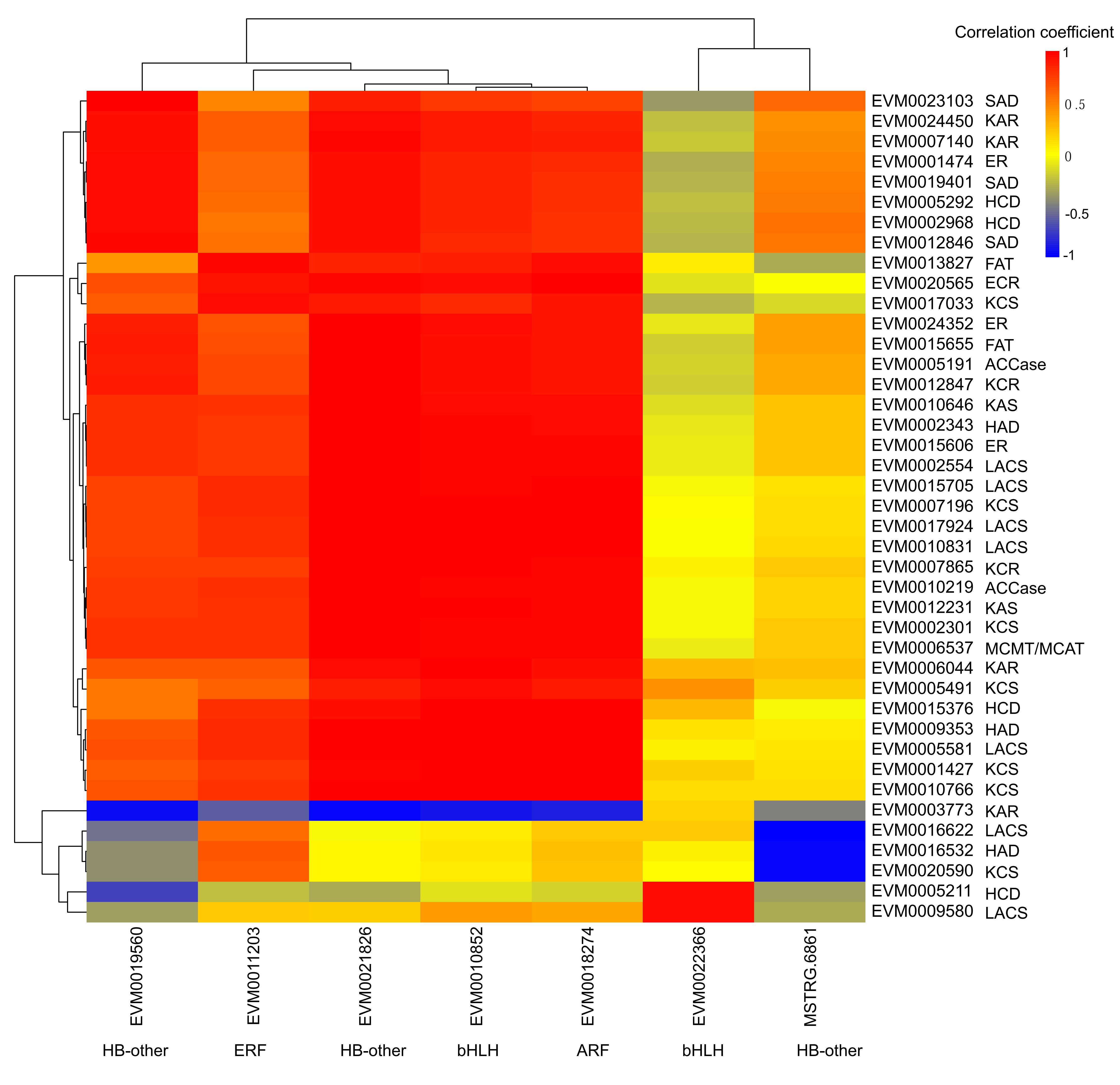

Supplement: Supplementary file 1 [file DataSheet1.ZIP › supplementary figures and tables/Figure S5.jpg]

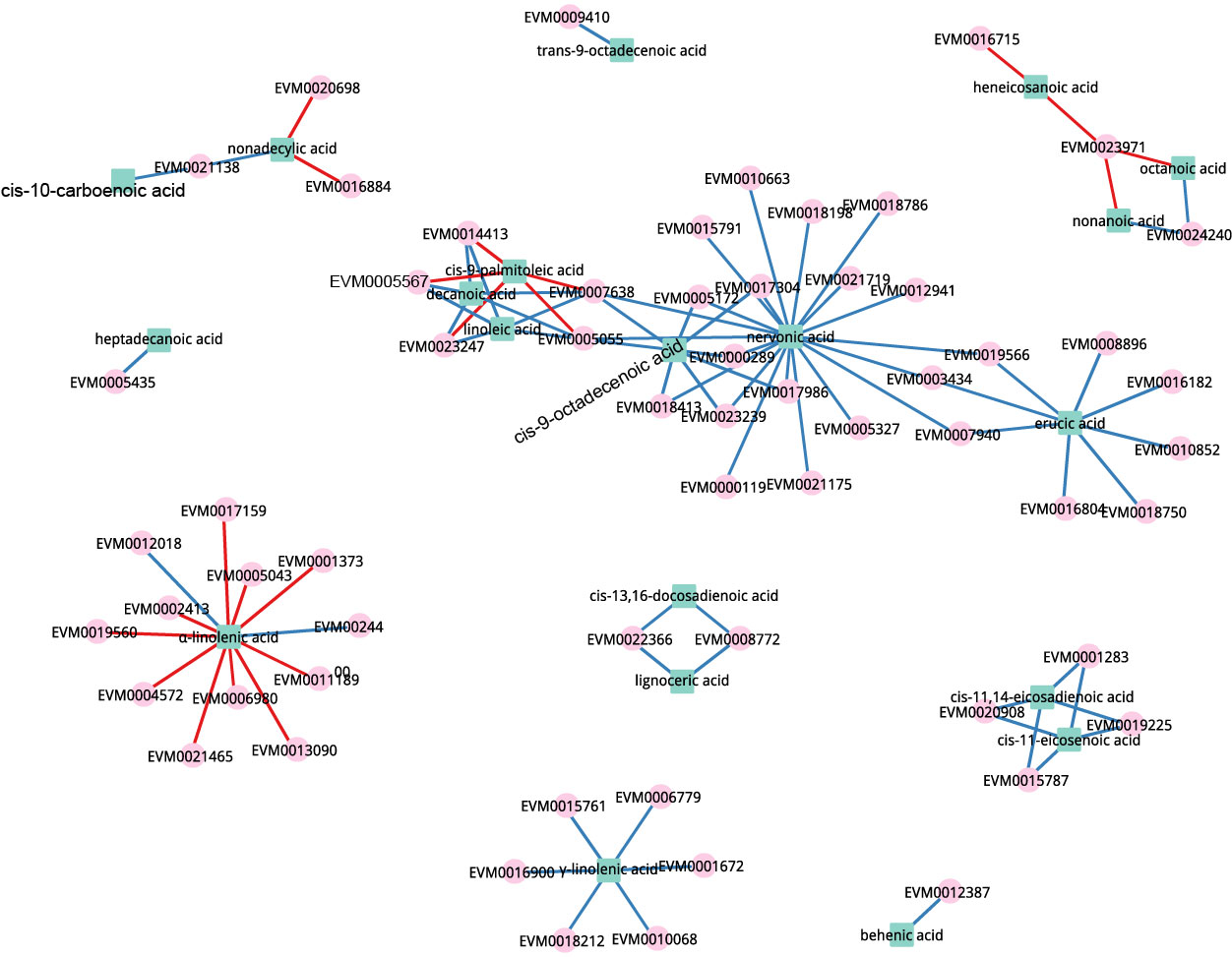

Supplement: Supplementary file 1 [file DataSheet1.ZIP › supplementary figures and tables/Figure S6.jpg]

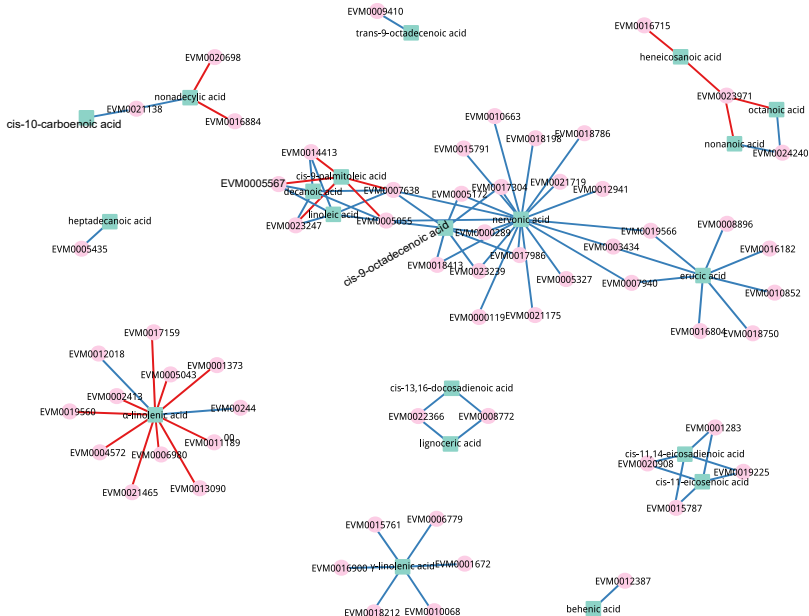

Supplement: Supplementary file 1 [file DataSheet1.ZIP › supplementary figures and tables/Figure S6.pdf]
